# Non-Specialist Psychosocial Interventions for Children and Adolescents with Intellectual Disability or Lower-Functioning Autism Spectrum Disorders: A Systematic Review

**DOI:** 10.1371/journal.pmed.1001572

**Published:** 2013-12-17

**Authors:** Brian Reichow, Chiara Servili, M. Taghi Yasamy, Corrado Barbui, Shekhar Saxena

**Affiliations:** 1 Yale Child Study Center, New Haven, Connecticut, United States of America; 2 University of Connecticut Health Center, Farmington, Connecticut, United States of America; 3 World Health Organization, Geneva, Switzerland; 4 WHO Collaborating Centre for Research and Training in Mental Health and Service Evaluation, Section of Psychiatry, Department of Public Health and Community Medicine, University of Verona, Verona, Italy; Association for the Mentally Challenged, India

## Abstract

**Background:**

The development of effective treatments for use by non-specialists is listed among the top research priorities for improving the lives of people with mental illness worldwide. The purpose of this review is to appraise which interventions for children with intellectual disabilities or lower-functioning autism spectrum disorders delivered by non-specialist care providers in community settings produce benefits when compared to either a no-treatment control group or treatment-as-usual comparator.

**Methods and Findings:**

We systematically searched electronic databases through 24 June 2013 to locate prospective controlled studies of psychosocial interventions delivered by non-specialist providers to children with intellectual disabilities or lower-functioning autism spectrum disorders. We screened 234 full papers, of which 34 articles describing 29 studies involving 1,305 participants were included. A majority of the studies included children exclusively with a diagnosis of lower-functioning autism spectrum disorders (15 of 29, 52%). Fifteen of twenty-nine studies (52%) were randomized controlled trials and just under half of all effect sizes (29 of 59, 49%) were greater than 0.50, of which 18 (62%) were statistically significant. For behavior analytic interventions, the best outcomes were shown for development and daily skills; cognitive rehabilitation, training, and support interventions were found to be most effective for improving developmental outcomes, and parent training interventions to be most effective for improving developmental, behavioral, and family outcomes. We also conducted additional subgroup analyses using harvest plots. Limitations include the studies' potential for performance bias and that few were conducted in lower- and middle-income countries.

**Conclusions:**

The findings of this review support the delivery of psychosocial interventions by non-specialist providers to children who have intellectual disabilities or lower-functioning autism spectrum disorders. Given the scarcity of specialists in many low-resource settings, including many lower- and middle-income countries, these findings may provide guidance for scale-up efforts for improving outcomes for children with developmental disorders or lower-functioning autism spectrum disorders.

**Protocol Registration:**

PROSPERO CRD42012002641

*Please see later in the article for the Editors' Summary*

## Abstract 2

In a systematic review, Brian Reichow and colleagues assess the evidence that non-specialist care providers in community settings can provide effective interventions for children and adolescents with intellectual disabilities or lower-functioning autism spectrum disorders.

*Please see later in the article for the Editors' Summary*

## Editors' Summary

**Background:**

Newborn babies are helpless, but over the first few years of life, they acquire motor (movement) skills, language (communication) skills, cognitive (thinking) skills, and social (interpersonal interaction) skills. Individual aspects of these skills are usually acquired at specific ages, but children with a development disorder such as an autism spectrum disorder (ASD) or intellectual disability (mental retardation) fail to reach these “milestones” because of impaired or delayed brain maturation. Autism, Asperger syndrome, and other ASDs (also called pervasive developmental disorders) affect about 1% of the UK and US populations and are characterized by abnormalities in interactions and communication with other people (reciprocal socio-communicative interactions; for example, some children with autism reject physical affection and fail to develop useful speech) and a restricted, stereotyped, repetitive repertoire of interests (for example, obsessive accumulation of facts about unusual topics). About half of individuals with an ASD also have an intellectual disability—a reduced overall level of intelligence characterized by impairment of the skills that are normally acquired during early life. Such individuals have what is called lower-functioning ASD.

**Why Was This Study Done?:**

Most of the children affected by developmental disorders live in low- and middle-income countries where there are few services available to help them achieve their full potential and where little research has been done to identify the most effective treatments. The development of effective treatments for use by non-specialists (for example, teachers and parents) is necessary to improve the lives of people with mental illnesses worldwide, but particularly in resource-limited settings where psychiatrists, psychologists, and other specialists are scarce. In this systematic review, the researchers investigated which psychosocial interventions for children and adolescents with intellectual disabilities or lower-functioning ASDs delivered by non-specialist providers in community settings produce improvements in development, daily skills, school performance, behavior, or family outcomes when compared to usual care (the control condition). A systematic review identifies all the research on a given topic using predefined criteria; psychosocial interventions are defined as therapy, education, training, or support aimed at improving behavior, overall development, or specific life skills without the use of drugs.

**What Did the Researchers Do and Find?:**

The researchers identified 29 controlled studies (investigations with an intervention group and a control group) that examined the effects of various psychosocial interventions delivered by non-specialist providers to children (under 18 years old) who had a lower-functioning ASD or intellectual disability. The researchers retrieved information on the participants, design and methods, findings, and intervention characteristics for each study, and calculated effect sizes—a measure of the effectiveness of a test intervention relative to a control intervention—for several outcomes for each intervention. Across the studies, three-quarters of the effect size estimates were positive, and nearly half were greater than 0.50; effect sizes of less than 0.2, 0.2–0.5, and greater than 0.5 indicate that an intervention has no, a small, or a medium-to-large effect, respectively. For behavior analytic interventions (which aim to improve socially significant behavior by systematically analyzing behavior), the largest effect sizes were seen for development and daily skills. Cognitive rehabilitation, training, and support (interventions that facilitates the relearning of lost or altered cognitive skills) produced good improvements in developmental outcomes such as standardized IQ tests in children aged 6–11 years old. Finally, parental training interventions (which teach parents how to provide therapy services for their child) had strong effects on developmental, behavioral, and family outcomes.

**What Do These Findings Mean?:**

Because few of the studies included in this systematic review were undertaken in low- and middle-income countries, the review's findings may not be generalizable to children living in resource-limited settings. Moreover, other characteristics of the included studies may limit the accuracy of these findings. Nevertheless, these findings support the delivery of psychosocial interventions by non-specialist providers to children who have intellectual disabilities or a lower-functioning ASD, and indicate which interventions are likely to produce the largest improvements in developmental, behavioral, and family outcomes. Further studies are needed, particularly in low- and middle-income countries, to confirm these findings, but given that specialists are scarce in many resource-limited settings, these findings may help to inform the implementation of programs to improve outcomes for children with intellectual disabilities or lower-functioning ASDs in low- and middle-income countries.

**Additional Information:**

Please access these websites via the online version of this summary at http://dx.doi.org/10.1371/journal.pmed.1001572.

- This study is further discussed in a *PLOS Medicine* Perspective by Bello-Mojeed and Bakare
- The US Centers for Disease Control and Prevention provides information (in English and Spanish) on developmental disabilities, including autism spectrum disorders and intellectual disability
- The US National Institute of Mental Health also provides detailed information about autism spectrum disorders, including the publication “A Parent's Guide to Autism Spectrum Disorder”
- Autism Speaks, a US non-profit organization, provides information about all aspects of autism spectrum disorders and includes information on the Autism Speaks Global Autism Public Health Initiative
- The National Autistic Society, a UK charity, provides information about all aspects of autism spectrum disorders and includes personal stories about living with these conditions
- The UK National Health Service Choices website has an interactive guide to child development and information about autism and Asperger syndrome, including personal stories, and about learning disabilities
- The UK National Institute for Health and Care Excellence provides clinical guidelines for the management and support of children with autism spectrum disorders
- The World Health Organization provides information on its Mental Health Gap Action Programme (mhGAP), which includes recommendations on the management of developmental disorders by non-specialist providers; the mhGAP Evidence Resource Center provides evidence reviews for parent skills training for management of children with intellectual disabilities and pervasive developmental disorders and interventions for management of children with intellectual disabilities
- PROSPERO, an international prospective register of systematic reviews, provides more information about this systematic review

## Introduction

Developmental disorder is an umbrella term covering disorders such as intellectual disability/mental retardation as well as pervasive developmental disorders including autism spectrum disorders. Developmental disorders usually have a childhood onset, impairment or delay in functions related to central nervous system maturation, and a steady course that persists into adulthood. Intellectual disability, or mental retardation, is defined as “a condition of arrested or incomplete development of the mind, which is especially characterized by impairment of skills manifested during the developmental period, which contribute to the overall level of intelligence, i.e., cognitive, language, motor, and social abilities” [1]. Autism spectrum disorders (also called pervasive developmental disorders), such as autism, Asperger syndrome, childhood disintegrative disorder, and atypical autism, comprise a range of conditions characterized by a varied mixture of impaired capacity for reciprocal socio-communicative interaction and a restricted, stereotyped, repetitive repertoire of interests and activities. The level of intellectual functioning for individuals with autism spectrum disorders is extremely variable, extending from profound impairment to superior nonverbal cognitive skills. It is estimated that up to 50% of individuals with an autism spectrum disorder also have an intellectual disability [2].

Neurodevelopmental disorders, including intellectual disability and autism spectrum disorders, affect children worldwide, might be a more prevalent condition in lower- and middle-income countries (LMICs) compared to higher-income countries (HICs) [3], and account for more than 0.4% of all disability-adjusted life years [4]. Although most of the children and families affected by neurodevelopmental disorders live in developing countries, nearly all research, preventative efforts, and services are directed towards individuals living in the world's wealthiest countries [5]. International evidence has shown that 75% to 85% of individuals with mental disorders in some LMICs do not receive any treatment services [6], and the lack of services prevents children from realizing a high quality of life and increases the burden on families [4]. The World Health Organization's *Atlas: Global Resources for Persons with Intellectual Disabilities* [7] provides more evidence of these disparities by showing that available resources are proportional to a country's income. In LMICs significantly fewer resources are available to dedicate to providing mental health services, and specialized human resources to deliver interventions are often either not available or available at a much lower rate than in HICs [5],[7]–[11]. Additional barriers to increased service provision for childhood mental disorders in LMICs include the lack of evidence on effective treatments delivered in these settings and a limited capacity for identifying children with developmental disorders [9],[12].

The development of effective treatments for use by non-specialists (e.g., mental health care providers who are not psychiatrists, psychologists, or psychiatric nurse practitioners) is listed among the top research priorities for improving the lives of people with mental illness worldwide [13]. Task shifting approaches that build the capacities of a range of care providers in community settings have been successfully adopted and can be instrumental for increasing access to care for individuals with a range of mental disorders in low-resource settings [14],[15]. Evidence on the effectiveness of provision of psychosocial interventions for intellectual disabilities and autism spectrum disorders by non-specialist providers in HICs is emerging [16].

Previous systematic reviews on psychosocial interventions for intellectual disabilities and autism and other pervasive developmental disorders conducted in LMICs identified few relevant papers, and many of the studies had significant methodological shortcomings [17]–[19]. Thus, formulating practice guidance based solely on the findings from studies conducted in LMICs, which would provide the results with the best ecological validity, is difficult to accomplish at this time. Hence, there is a need to conduct a review that includes psychosocial interventions conducted in HICs that might be feasibly adapted for implementation in LMICs. The purpose of this review is to provide an appraisal of which interventions for children and adolescents with intellectual disabilities or lower-functioning autism spectrum disorders delivered by non-specialist care providers in community settings produce benefits in development, daily skills, school performance, behavior, or family outcomes when compared to either a no-treatment control group or treatment-as-usual comparator.

## Methods

### Selection Criteria

We included studies in our review meeting the following inclusion criteria. First, the study contained participants with neurodevelopmental disorders who, on average, had a full scale IQ<70 and were, on average, under the age of 18 y. Second, the study used a prospective controlled study design, specifically, a study design comparing a treatment to a control condition, regardless of randomization. We included non-randomized controlled studies because we thought we might have difficulty locating a large pool of randomized controlled trials from which to build recommendations, and because we wanted to locate all studies with high ecological validity (e.g., studies conducted in similar contexts and under similar conditions regardless of randomization). Third, the study involved a psychosocial intervention. For this review, psychosocial intervention was defined as a therapy, education, training, or support aimed at improving behavior, general overall development, or specific life skills without the use of psychopharmacologic agents. Fourth, an individual who was not a specialist provided the psychosocial intervention or parent education to the child or parents, respectively. Stated differently, we included only studies in which a non-specialist (e.g., teacher, aide, parent, general practitioner, nurse practitioner, or local clinician) provided the direct intervention sessions to the child or conducted the parent education sessions. For this review, we considered psychiatrists, psychiatric nurse practitioners, psychologists, speech and language pathologists, occupational therapists, and physical therapists, among others, to be specialist providers. Fifth, the study was published in English, French, or Spanish. Included and excluded studies were collected following Preferred Reporting Items for Systematic Reviews and Meta-Analyses (PRISMA) [20].

### Search Methods

We conducted an electronic database search of African Index Medicus, AFRO Library, the Cochrane Central Register of Controlled Trials, Cumulative Index to Nursing and Allied Health, Embase, Western Pacific Region Index Medicus, Literatura Latino-Americana e do Caribe em Ciências da Saúde, Medline, and PsycINFO through 24 June 2013 using the search strategies shown in Texts S1, S2, S3, S4, S5. Because some of our inclusion criteria, mainly the inclusion of non-randomized studies, have the potential to increase bias, we decided not to search gray literature, which has also been considered to potentially introduce additional bias by not providing a representative sample of studies, by containing studies of lower quality, and by having more favorable results being provided more readily [21]. All titles and abstracts were initially screened by one author in order to exclude clearly irrelevant articles, and two authors, working independently and in duplicate, screened full papers of potentially relevant articles and determined which studies met all inclusion criteria. After the database search, we examined the reference lists of ten recent reviews of psychosocial interventions for individuals with developmental disabilities [17]–[19],[22]–[28] for possible studies that were not located in the database search.

### Variable Definitions and Coding

We coded 16 variables related to research methods, participant characteristics, treatment characteristics, and study results. All variables and effect size estimates were coded independently by two abstractors, and all discrepancies were resolved through mediation.

We coded four variables related to the participants of each study. First, we coded child diagnosis by indicating whether (a) all participants had a diagnosis of a lower-functioning autism spectrum disorder, (b) all participants had intellectual disability without an autism spectrum disorder, or (c) there was a mixture of participants with diagnoses of intellectual disability with an autism spectrum disorder and participants with intellectual disability without an autism spectrum disorder. Second, we coded the sample size by recording the total number of participants in the study and the number of participants in the treatment and comparison groups. Third, we calculated the mean age of the participants and provided a standard deviation and/or range when possible. Fourth, we estimated the participant's skill level by recording the mean (and standard deviation) of an IQ or developmental quotient (DQ) when it was provided in the study report or by calculating a DQ by dividing the mean mental age by mean chronological age and multiplying by 100. When only a range was reported, e.g., IQ<70, the range was recorded and reported instead of a mean and standard deviation.

We coded five variables related to the research study design and methods. First, we coded whether the study used a randomized controlled trial or quasi-experimental research design. Second, we identified the country in which the study occurred (i.e., study location). Third, within the study location, we coded whether the study occurred in a country classified by the World Bank in July 2012 as Lower Income (gross national income <US$1,025), Lower-Middle Income (US$1,026–US$4,035), Upper-Middle Income (US$4,036–US$12,475), or High Income (US$12,476 or more). Fourth, for outcomes, we classified the measures reported in each study into one of five categories: (a) development (e.g., standardized tests of IQ, developmental progress, or language; measures of cognitive processes), (b) daily skills (e.g., adaptive behavior), (c) school performance (e.g., reading ability, literacy skills), (d) behavior (e.g., problem behavior, symptoms of behavioral disorders such as hyperactivity and inattention), or (e) family (e.g., parental stress, parental sense of competence, parenting skills). Fifth, we evaluated risk of bias using an adaptation of the Cochrane Collaboration's Risk of Bias Tool [29] to assess eight items: sequence generation, allocation concealment, performance bias, detection bias, attrition, selective outcome reporting, protection against contamination, and baseline imbalance. The adaptations to the Cochrane Collaboration's Risk of Bias Tool were made to accommodate our decision to include non-randomized trials and are consistent with emerging recommendations of the Cochrane Collaboration's Non-Randomised Studies Methods Group [30],[31].

We coded two variables related to study results and findings. First, for the effect size, we calculated the standardized mean difference between the posttreatment means of the treatment and comparison groups using Cohen's *d* for each outcome category, using effect size calculators based on the formulae provided in Lipsey and Wilson [32] housed on the Campbell Collaboration website (http://www.campbellcollaboration.org/resources/effect_size_input.php). Cohen's *d* [33] is an effect size reported as standard deviations (i.e., *d* = 1.0 is a one-standard-deviation difference between treatment and control) that is calculated by dividing the difference in mean outcome between groups by the standard deviation of outcomes among participants. Classic guidelines [33] for interpreting the magnitude of effect for Cohen's *d* suggest that an effect size <0.20 indicates no effect, 0.20–0.50 indicates a small effect, 0.50–0.80 indicates a medium effect, and >0.80 indicates a large effect. We have chosen to combine the medium and large effects in our interpretation of the magnitude of effect to reflect that many clinically significant effects can be found in effect sizes less than 0.80 but greater than 0.50. The statistical significance of effect sizes was determined by examination of the 95% CI when available. For outcomes for which effect sizes were averaged, statistical significance is indicated only if the 95% CI for all measures indicated statistical significance. When more than one outcome was included in a single study for one of our outcome categories (e.g., two separate family measures such as parent stress and quality of life), we calculated an unweighted mean of all variables within the outcome category and provided the range of effect size estimates instead of the standard deviation and 95% confidence intervals. We chose to present our effect size estimates for studies with multiple measures within one outcome category using this method because measures of the same construct within one sample are likely to be highly correlated, which can have significant impacts on mean calculations that are used to produce more standard estimates that include standard deviations and confidence intervals [34]. When multiple treatment groups receiving similar interventions were reported in one study, we averaged the posttreatment means and standard deviations for all treatment groups and compared this to the posttreatment scores from the control group. We considered synthesizing results statistically using methods such as meta-analysis, including multiple regression techniques, but decided against such analyses given the small number of studies that were ultimately located, and our decision to include randomized and non-randomized trials. Second, in addition to the effect size, we coded and reported the results of statistical significance testing, as reported in the study article, including, when available, a description of differential effects.

We coded five intervention characteristic variables. First, we determined the type of psychosocial intervention, which we categorized into three categories: (a) behavior analytic interventions, (b) cognitive rehabilitation, training, and support, or (c) parent training interventions. Second, the first author created a summary of intervention methods, which is reported as the intervention description. Included in this description, at a minimum, is an indication of whether the treatment was delivered in an individual or group format (if group, the number of participants per group is provided), the location where the treatment or training sessions were conducted, and whether the intervention contained a parent component (behavior analytic and cognitive rehabilitation only). Third, we coded a variable describing who provided the treatment to the child or the training to the parents (intervention agent, training, and supervision). Within this variable, when provided in the study report, we quantified the amount of training and supervision received by the treatment provider or parent trainer prior to and/or during the course of each study. Fourth, we quantified the intervention density by recording, if provided, the duration of each session, the number of sessions per week, and the number of weeks of treatment. Fifth, we calculated the total hours of therapy each participant received, on average, using the intervention density data we collected.

### Harvest Plots

To examine differential treatment effects across different variables of interest (e.g., study location, participant characteristics, treatment location, treatment/training provider, treatment density), we chose to construct graphical representations of the effect size using harvest plots [35],[36]. A harvest plot is a graphical display of treatment effects plotted across multiple variables, allowing for visual analysis of differential treatment effects. We chose to use harvest plots to analyze and synthesize the evidence because we determined that meta-analytic methods were not appropriate because of the large variability in intervention techniques and outcome variables.

In each harvest plot we have grouped the marks (the rectangles representing study effects) on the horizontal axis according to effect size estimates corresponding to Cohen's guidelines [33] (no effect, effect size <0.20; small effect, effect size = 0.20–0.49; and medium to large effect, effect size >0.50). The marks are grouped on the vertical axis by the type of psychosocial intervention (behavior analytic interventions; cognitive rehabilitation, training, and support; and parent training) that was used in each study. The heights of the marks represent the research methodology used in the study, with the taller marks indicating studies using randomized controlled trials and the shorter marks indicating studies using quasi-experimental designs. Statistical significance (determined by examination of the 95% CI when available) is indicated in the harvest plots for each study outcome by an asterisk above the mark. The marks are also color-coded by outcome category (black = development, gray = daily skills, white = school performance, vertical stripes = behavior, and horizontal stripes = family). We have indicated participant diagnostic categories for each study using black bars that are placed above the marks. Participant diagnostic characteristics (i.e., all participants had intellectual disability, all participants had lower-functioning autism spectrum disorder, or the study contained a mixture of participants with both intellectual disability and lower-functioning autism spectrum disorder) are indicated above each mark. Finally, all harvests plots contain multiple panels, which are labeled above each plot (e.g., comparison of results between outcomes, comparison between levels of cognitive impairment).

## Results

### Search Results

**Figure 1:**
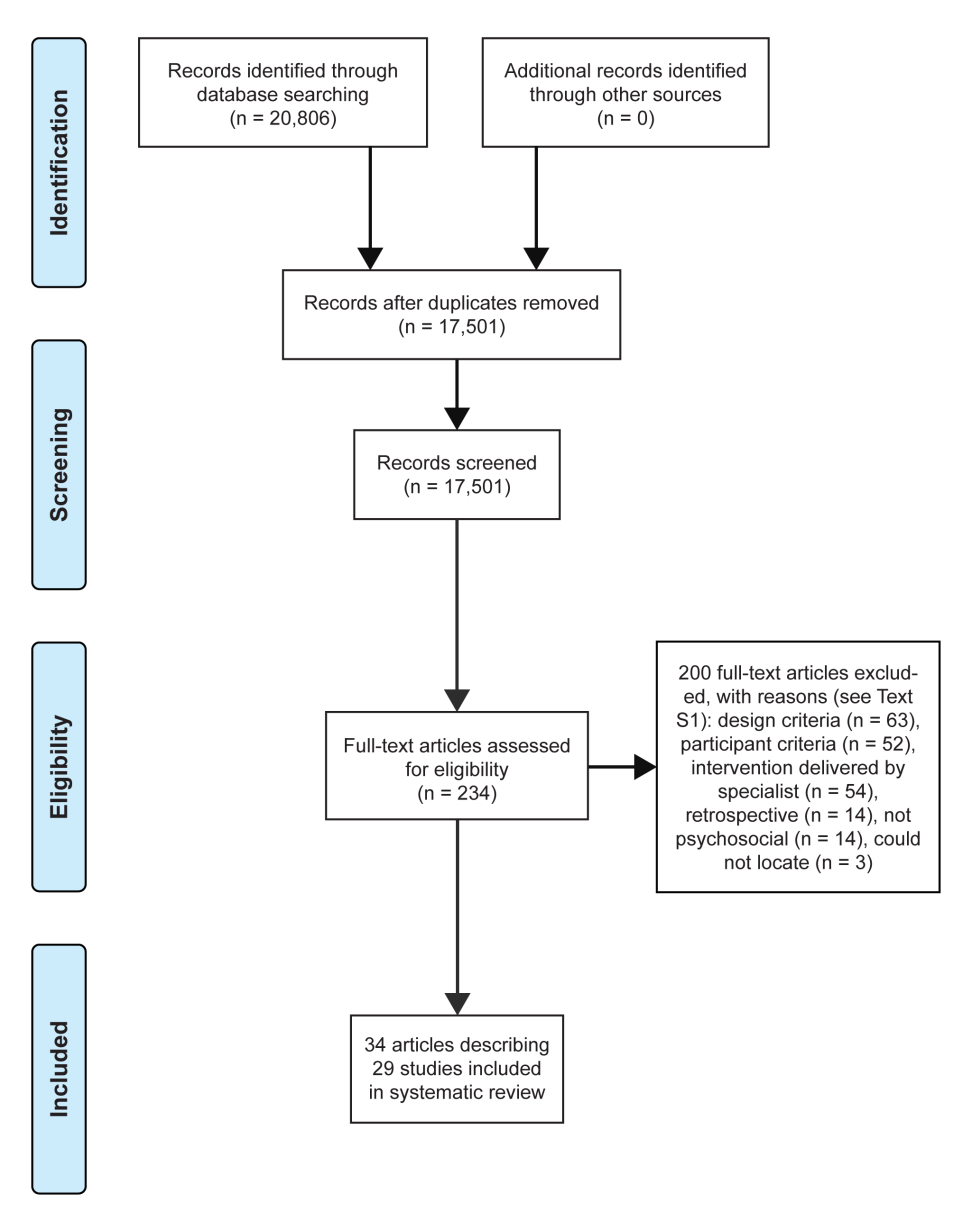
Study inclusion decision tree (using PRISMA flow diagram [20]).

We located 20,806 records; 17,501 records remained after deduplication. Two hundred thirty-four articles remained after the first author screened the titles and abstracts. The first and second authors examined the 234 full papers independently, and in duplicate, for inclusion in this review. Our database search located 34 articles describing 29 studies that met all inclusion criteria. The primary reasons for exclusion are provided in Text S6 and are shown in the PRISMA flow diagram of Figure 1. No additional studies were located in the hand search of previous reviews. We have used study as the unit of analysis for all results to ensure that studies reported in multiple articles are not weighted more heavily than those presented in one article.

### Participant Characteristics

Table 1 presents the participant characteristics of the 1,305 participants across the 29 studies included in this review. The mean age of the participants ranged from 0.4 y (about 5 mo) to 15.8 y. A majority of the studies (19 of 29, 66%) had samples with a mean age less than 6 y; nine of 29 (31%) studies had a mean age of participants older than 6 y (a mean age was not able to be obtained for one study, 3%). Across studies, a majority of the studies included children exclusively with a diagnosis of a lower-functioning autism spectrum disorder (15 of 29, 52%); six of 29 (21%) studies included a mixture of participants with intellectual disability or lower-functioning autism spectrum disorder, and eight of 29 studies (28%) included participants who had intellectual disability without an autism spectrum disorder. Sixteen of 29 studies (55%) had IQ or DQ estimates between 50 and 69, and five of 29 (17%) studies had participants with IQ or DQ estimates below 50 (a mean IQ or DQ could not be obtained for eight of 29 studies, 28%).

**Table 1:** Included studies and participant characteristics.

First Author and Year of Publication of Original Study [Reference]	Child Diagnosis	*n* (T, C)^a^	Age (Years) (SD; Range)	Mean (SD) Skill Level
**Behavior analytic techniques**				
Kaale 2012 [66]	ASD	51 (34, 27)	4.1 (0.7; 2.0–5.0)	DQ: 56.6 (19.5)
Dawson 2010 [61],[71]	ASD	45 (24, 21)	2.0 (0.3; 1.5–2.5)	IQ: 60.2 (8.9)
Smith 2000 [70]	ASD	28 (15, 13)	2.9 (0.5; 1.5–3.5)	IQ: 50.6 (12.6)
Jocelyn 1998 [65]	ASD	35 (16, 19)	3.6 (0.8; 2.0–6.0)	IQ: 62.8 (27.5)
Eikeseth 2012 [63]	ASD	59 (35,24)	4.1 (2.1; 2.0–7.3)	DQ: 48.2 (n/a)
Peters-Scheffer 2010 [67]	ASD	34 (12, 22)	4.5 (0.7; 3.0–6.0)	DQ: 46.4 (13.2)
Eikeseth 2007 [62],[72]	ASD	25 (13, 12)	5.5 (0.9; 4.0–7.0)	IQ: 63.6 (13.2)
Reed 2007 [68]	ASD	32 (12, 20)	3.5 (n/a; 2.7–3.9)	IQ: 52.9 (6.3)
Remington 2007 [69],[73]	ASD	44 (23, 21)	3.1 (0.4; 2.5–3.5)	IQ: 61.9 (16.5)
Cohen 2006 [60]	ASD	42 (21, 21)	2.7 (0.4; 1.5–3.5)	IQ: 60.5 (15.6)
Howard 2005 [64]	ASD	45 (29, 16)	2.9 (0.5; 0–4.0)	IQ: 59.2 (16.6)
**Cognitive rehabilitation, training, and support**				
Browder 2012 [76],[85]	ASD, ID	93 (47, 46)	n/a (n/a; 8.0–11.0)	IQ: 42.5 (13.0)
Burgoyne 2012 [77]	ID	57 (29, 28)	6.6 (1.3; 5.0–10.0)	IQ: <70^b^ (n/a)
Allor 2010 [75],[84]	ASD, ID	59 (34, 25)	7.9 (1.5; n/a)	IQ: range 40–69
Elwan 2010 [78] c	ID	40 (10, 10, 10, 10)^d^	4.9 (0.6; n/a)	IQ: 60 (estimated)
Panerai 2009 [81]	ASD	23 (13, 10)	8.9 (2.1; n/a)	DQ: 20.9 (n/a)
Goetz 2008 [79]	ID	15 (8, 7)	10.2 (n/a; 8.0–12.0)	IQ: <70^b^ (n/a)
Perez 2008 [82]	ID	113 (63, 50)	n/a (n/a; 11–16)	IQ: 52.7 (10.5)
Tsang 2007 [83] c	ASD	34 (18, 16)	4.1 (0.6; 3.0–5.9)	IQ: 67.1 (14.6)
Jespen 2002 [80]	ASD, ID	46 (23, 23)	15.8 (n/a; 14.0–16.0)	IQ: 57 (1.1)
**Parent training interventions**				
Wong 2010 [94] c	ASD	17 (9, 8)	2.2 (0.5; 1.4–3.0)	DQ: 67.4 (n/a)
Shin 2009 [91] c	ID	20 (6, 14)	4.5 (1.0; 3.0–6.0)	IQ: <70^b^ (n/a)
Plant 2007 [88]	ASD, ID	74 (26, 24, 24)^e^	4.6 (1.1; 0–6)	IQ: <70^b^ (n/a)
Del Giudice 2006 [86]	ID	32 (21,11)	0.4 (0.2; n/a)	DQ: 54.3 (22.6)
Roberts 2006 [89]	ASD, ID	32 (17, 15)	4.3 (1.0; n/a)	IQ: 62.5 (16.6)
Russell 1999 [90] c	ID	52 (26, 26)	6.4 (2.7; 0–13.0)	IQ: <70^b^ (n/a)
Varma 1992 [93] c	ID	80 (40, 40)	7.3 (2.3; 3.0–10.0)	IQ: 49.9 (10.6)
McConachie 2005 [87]	ASD, ID	51 (26, 25)	3.1 (0.6; 2.0–4.0)	IQ: <70^b^ (n/a)
Shu 2005 [92]	ASD	27 (8, 19)	Not reported	Not reported

DQ calculated as mean mental age/mean chronological age × 100.

^a^ Total number of children in study (*n*) and the number of children in the treatment (T) and control (C) groups.

^b^ IQ estimate based on our interpretation of participant characteristics.

^c^ LMIC status according to the World Bank.

^d^ This study contained three treatment groups and one control group (*n* = 10).

^e^ This study contained two treatment groups and one control group (*n* = 24).

ASD, autism spectrum disorder; ID, intellectual disability; n/a, not available; SD, standard deviation.

### Intervention Characteristics

Tables 2–4 provide information on the intervention characteristics across types of psychosocial intervention for studies using behavior analytic techniques; cognitive rehabilitation, training, and support; and parent training interventions, respectively. The treatment agents (e.g., therapists) typically delivered interventions with multiple hours of treatment per week (range 1 to 40 h per week). Treatment duration for the behavior analytic studies and cognitive rehabilitation, training, and support studies was often long, lasting, in many cases, over 100 wk (range 3 to 156 wk); the duration of parent training interventions was typically much shorter, with most lasting between 8 and 12 wk. As seen in the intervention description, a variety of methods and curricula were used across studies. Given that the behavior analytic and cognitive rehabilitation, training, and support studies had higher weekly treatment densities and longer treatment durations, it is not surprising that the number of total contact hours was higher in these studies (range 18 to 6,240 h) than in studies of parent training interventions (range 5 to 52 h). Table 5 provides an overview of the intervention agents, with training and supervision requirements, across all studies. There were multiple types of non-specialist providers including teachers (*n* = 12), aides (*n* = 10), and community therapists/clinicians (*n* = 6). A majority of the studies reported that training and/or ongoing supervision of the non-specialist providers occurred, albeit often with little specificity. When reported, the frequency and duration of training and supervision varied highly across studies from “ongoing on-the-job training” to over 40 h of initial training before beginning to deliver treatment.

**Table 2:** Intervention description, intervention density, and total hours of intervention for behavior analytic intervention studies.

First Author and Year of Publication of Original Study [Reference]	Child Diagnosis	Intervention Description	Intervention Density	Total Hours of Treatment
Kaale 2012 [66]	ASD	Joint-attention training based on Kasari et al. [102] combining developmental and behavioral treatment approaches delivered by a teacher in a school setting during 1∶1 instruction	Ten 20-min sessions per week for 8 wk	27 h
Dawson 2010 [61],[71]	ASD	A developmentally sequenced behavioral treatment based on the Early Start Denver Model [103] delivered in a 1∶1 instructional format by a therapist in the child's home with ongoing parent involvement	Ten 2-h sessions per week; M = 15.2 (SD = 1.4) h per week for 123 (SD = 14.6) wk	1,870 h
Smith 2000 [70]	ASD	Intensive applied behavior analysis based on Lovaas [104] model providing intensive behavioral treatment delivered mostly in a 1∶1 instructional format by instructional aides in the child's home, with ongoing parent involvement	18–31 h per week (M = 24.5, SD = 3.7) for M = 143.8 (SD = 47.3) wk	3,523 h
Jocelyn 1998 [65]	ASD	Caregiver-based intervention program delivered in 1∶1 format in child care settings by providers who received training on behavioral principles in behavior management with an additional parent component	M = 21.4 h per week for 12 wk	257 h
Eikeseth 2012 [63]	ASD	Applied behavior analysis based on Lovaas [104],[105] manuals providing intensive behavioral treatment delivered mostly in a 1∶1 instructional format by instructional aides in a school setting with ongoing parent involvement	15–37 h per week (M = 23.0) for 52 wk	1,196 h
Peters-Scheffer 2010 [67]	ASD	Low-intensity behavior analytic intervention delivered in a school setting by an instructional aide in a 1∶1 instructional format including additional parent training	5–10 h per week (M = 6.3) for 34 wk	214 h
Eikeseth 2007 [62],[72]	ASD	Applied behavior analysis based on Lovaas [104],[105] manuals providing intensive behavioral treatment delivered mostly in a 1∶1 instructional format by instructional aides in the child's home with ongoing parent involvement	18–28 h per week for 135 wk	2,430–3,780 h
Reed 2007 [68]	ASD	Intensive behavior analytic intervention [104],[106],[107] provided mostly in a 1∶1 instructional format by instructional aides in the child's home	20–40 h per week (M = 30.4) for 43 wk	1,307 h
Remington 2007 [69],[73]	ASD	Intensive behavior analytic intervention based on Green et al. [108] manual providing intensive behavioral treatment delivered mostly in a 1∶1 instructional format by instructional aides in the child's home with ongoing parent involvement	18–30 h per week; M = 25.6 (SD = 4.8) h of treatment per week for 104 wk	2,662 h
Cohen 2006 [60]	ASD	Applied behavior analysis based on Lovaas [105] manual providing intensive behavioral treatment delivered mostly in a 1∶1 instructional format by instructional aides in the child's home with ongoing parent involvement	35–40 h per week for 156 wk	5,460–6,240 h
Howard 2005 [64]	ASD	Applied behavior analysis based on treatment programs described in the manuals of Maurice et al. [109],[110] providing intensive behavioral treatment delivered mostly in a 1∶1 instructional format by instructional aides in the child's home with ongoing parent involvement	25–40 h per week for 61 wk	1,525–2,440 h

No behavior analytic intervention studies were conducted in LMICs (LMIC status per World Bank).

ASD, autism spectrum disorder; ID, intellectual disability; M, mean; SD, standard deviation.

**Table 3:** Intervention description, intervention density, and total hours of intervention for cognitive rehabilitation, training, and support intervention studies.

First Author and Year of Publication of Original Study [Reference]	Child Diagnosis	Intervention Description	Intervention Density	Total Hours of Treatment
Browder 2012 [76],[85]	ASD, ID	Early Literacy Skills Builder [111] curriculum delivered in small groups (size 2–4) delivered in schools using behavioral strategies including response-prompting techniques	Five 20-min sessions per week for 30 wk	50 h
Burgoyne 2012 [77]	ID	Multicomponent phonics-based reading program with language instruction delivered in a 1∶1 instructional format	Five 40-min sessions per week for 40 wk	133 h
Allor 2010 [75],[84]	ASD, ID	300 direct instruction small group instructional sessions delivered in the school targeting reading based on Early Interventions in Reading [112]	Five 40–50-min sessions per week for 60 wk	200–250 h
Elwan 2010 [78] a	ID	T1: integration of child with disability with small group of peers without disabilities in school; T2: cognitive training during 1∶1 instruction in school; T3: T1 and T2	T1: 2 h per day 3 d per week for 3 wk; T2: eight 1-h sessions for 3 wk	T1: 18 h; T2: 24 h
Panerai 2009 [81]	ASD	Full-time schooling incorporating principles of TEACCH program [113] delivered in schools including daily 1∶1 instruction and an additional parent component (natural setting TEACCH group used for analyses, not residential)	Treatment provided during school day for 156 wk	5,460 h
Goetz 2008 [79]	ID	Phonological awareness intervention based on Jolly Phonics [114] and reading intervention [115] with additional speech-based component delivered during 1∶1 instruction in school	Five 40-min sessions per week for 8 wk	27 h
Perez 2008 [82]	ID	Special needs curriculum based on Gardner's multiple intelligences [116] and Anderson and Krathwohl's revision of Bloom's taxonomy [117], which provided students with individualized instruction in school	Treatment (curricula) used 4 h per week for one school year (about 40 wk)	160 h
Tsang 2007 [83] a	ASD	Full-time schooling incorporating principles of TEACCH program [113] delivered in school with 6–8 students with emphasis on visual structure and schedules as well as use of individualized work systems	7 h per day for 26 wk	910 h
Jespen 2002 [80]	ASD, ID	Cognitive education program involving individual, small group, and whole class lessons in school in which cognitive functions and strategies were mediated by teachers seeking to relate these functions to the student's everyday environments and routines	Treatment (curricula) used for 1 h per week for one school year (about 40 wk)	40 h

^a^ LMIC status according to the World Bank.

ASD, autism spectrum disorder; ID, intellectual disability; T1, T2, T3, treatment groups for studies with multiple treatments; TEACCH, Treatment and Education of Autistic and Related Communication Handicapped Children.

**Table 4:** Intervention description, intervention density, and total hours of intervention for parent training intervention studies.

First Author and Year of Publication of Original Study [Reference]	Child Diagnosis	Intervention Description	Intervention Density	Total Hours of Treatment
Wong 2010 [94] a	ASD	Clinic-based individual parent training using the Autism 1-2-3 program, which teaches parents techniques for increasing their child's eye contact, gestures, and vocalizations	Five 0.5-h sessions per week for 2 wk	5 h
Shin 2009 [91] a	ID	Individual in-home parent training based on the Portage curriculum [118] training parents to work with their children in the absence of professional resources	One 1-h session per week for 52 wk	52 h
Plant 2007 [88]	ASD, ID	T1: clinic-based individual parent training using Stepping Stones Triple P [119] model teaching parents how to promote development and manage behavioral problems; T2: T1 plus six enhanced training sessions	T1: one 1–1.5-h session per week for 10 wk; T2: one 1–1.5-h session per week for 16 wk	T1: 10–15 h; T2: 16–24 h
Del Giudice 2006 [86]	ID	Individual developmentally based training for parents of children with Down syndrome emphasizing 26 developmental sequences	About one session per month for 52 wk	Not specified
Roberts 2006 [89]	ASD, ID	Clinic-based individual parent training using the Stepping Stones Triple P [120] model instructing parents in how to identify causes of behavior problems and manage problem behavior while encouraging child development; included home visits	One 2-h session per week for 10 wk	20 h
Russell 1999 [90] a	ID	Clinic-based group interactive psychoeducation teaching parents about Down syndrome and intellectual disability, raising a child with disability, developmental milestones, and behavioral treatment methods	Two 1-h sessions per week for 10 wk	20 h
Varma 1992 [93] a	ID	Clinic-based individual parent training focusing on teaching parents broad information about intellectual disabilities and how to deliver behavioral modification in home settings	About one 1-h session per week for 3 mo	12 h
McConachie 2005 [87]	ASD, ID	Clinic-based parent training delivered in groups of eight based on the More Than Words [121] curriculum, which teaches parents techniques for facilitating social interaction and communication with their child; included home visits	One 2.5-h session per week for 8 wk	24 h
Shu 2005 [92]	ASD	Clinic-based group (size eight) support for mothers of children with intellectual disability focused on teaching the mothers how to handle stressors commonly associated with raising a child with a disability	One 90-min session per week for 10 wk	15 h

^a^ LMIC status according to the World Bank.

ASD, autism spectrum disorder; ID, intellectual disability; T1, T2, treatment groups for studies with multiple treatments.

**Table 5:** Intervention agent and training and supervision for included studies.

First Author and Year of Publication of Original Study [Reference]	Child Diagnosis	Agent	Training and Supervision
**Behavior analytic techniques**			
Kaale 2012 [66]	ASD	Teacher	1 d training and weekly supervision
Dawson 2010 [61],[71]	ASD	Therapist	2 mo training and weekly supervision
Smith 2000 [70]	ASD	Aide	Ongoing training and supervision (type and density not specified)
Eikeseth 2012 [63]	ASD	Aide	Ongoing training through 2 h per week supervision
Peters-Scheffer 2010 [67]	ASD	Aide	Workshop training (density not specified) and monthly supervision
Eikeseth 2007 [62],[72]	ASD	Aide	Ongoing training through 10 h per week supervision
Reed 2007 [68]	ASD	Aide	Ongoing training and supervision (type and density not specified)
Remington 2007 [69],[73]	ASD	Aide	Ongoing training (type and density not specified) and monthly supervision
Cohen 2006 [60]	ASD	Aide	Ongoing training and supervision (type and density not specified)
Howard 2005 [64]	ASD	Aide	Ongoing training and supervision (type and density not specified)
Jocelyn 1998 [65]	ASD	Teacher	15 h training (five 3-h workshops) and 3 h per week supervision
**Cognitive rehabilitation, training, and support**			
Browder 2012 [76],[85]	ASD, ID	Teacher	2 d training and ongoing supervision
Burgoyne 2012 [77]	ID	Teacher	5 d training and quarterly supervision
Allor 2010 [75],[84]	ASD, ID	Teacher	9 d training and monthly supervision
Elwan 2010 [78] a	ID	Teacher	Not specified
Panerai 2009 [81]	ASD	Aide	Not specified
Goetz 2008 [79]	ID	Aide	2 d training and supervision every other month
Perez 2008 [82]	ID	Teacher	Teacher was trained, but type and density of training not specified
Tsang 2007 [83] a	ASD	Teacher	Teacher was trained, but type and density of training not specified
Jespen 2002 [80]	ASD, ID	Teacher	3 d training and weekly supervision
**Parent training interventions**			
Wong 2010 [94] a	ASD	Therapist	Training provided, but type and density not specified
Shin 2009 [91] a	ID	Teacher	3 mo of weekly training and supervision every 3 wk
Plant 2007 [88]	ASD, ID	Practitioner	2 d training and weekly supervision
Del Giudice 2006 [86]	ID	Local therapists	Teacher was trained, but type and density of training not specified
Roberts 2006 [89]	ASD, ID	Teacher, SLP, OT, or psychologist	40 h training and ongoing supervision
Russell 1999 [90] a	ID	Special educator or psychologist	Not specified
Varma 1992 [93] a	ID	Local clinician	Not specified
McConachie 2005 [87]	ASD, ID	Local clinicians	Training provided by Hanen Centre (density not specified) with one supervision visit
Shu 2005 [92]	ASD	Nurse (training and supervision not specified)	Not specified

^a^ LMIC status according to the World Bank.

ASD, autism spectrum disorder; ID, intellectual disability; OT, occupational therapist; SLP, speech and language pathologist.

### Research Characteristics

Tables 6–8 show the research characteristics for studies using behavior analytic techniques; cognitive rehabilitation, training, and support; and parent training interventions, respectively. Just over half of the studies (15 of 29, 52%) used randomized controlled trial designs; prospective controlled study designs were used in 14 studies. Western Europe (12 of 29, 41%) and North America (United States and Canada; eight of 29, 28%) were the most common locations of the research studies included in this review. Of the remaining nine studies, six (21%) were conducted in Asia, two (7%) in Australia, and one (3%) in Africa. Six studies (21%) were conducted in countries classified by the World Bank as LMICs; each of these studies was conducted in a country classified as Lower-Middle Income. A majority of the studies conducted in LMICs examined parent training interventions. There was great variability with respect to the number and types of outcomes measured across studies. Within our outcome categories, some studies measured only one outcome category, while other studies measured up to four outcome categories (no study measured all five outcome categories). Developmental and daily skill outcomes were measured more frequently in psychosocial interventions utilizing behavior analytic techniques and cognitive rehabilitation, while family outcomes were measured more frequently in parent training programs. There was also great variability with respect to the measurement instruments used within each outcome category. For instance, developmental outcomes were measured using a number of different standardized assessments (e.g., developmental outcomes were measured using developmental assessments [37]–[39], standardized IQ tests [38],[40]–[47], and standardized language tests [48]–[58]), while daily skills were mostly measured using a single measure (i.e., Vineland Adaptive Behavior Scale [59]). Additional details on the measures and assessments used in each study by outcome type can be found in Table S1.

**Table 6:** Research design, results, and effect sizes for behavior analytic intervention studies.

First Author and Year of Publication of Original Study [Reference]	Child Diagnosis	Design	Results by Outcome	Effect Size by Outcome
Kaale 2012 [66]	ASD	RCT	Intervention group had significantly higher levels of joint attention and joint engagement [57] (development)	Range *d* = −0.31 to 0.57 (M = 0.16)
Dawson 2010 [61],[71]	ASD	RCT	Intervention group had significantly higher scores at follow-up for the developmental outcome [37] (a) and daily skills [59] (b), but no difference in restricted and repetitive behavior [122] (c)	(a) *d* = 0.60 (95% CI 0.00–1.20), (b) *d* = 0.73 (95% CI 0.13–1.34), (c) *d* = 0.36 (95% CI −0.23 to 0.95)
Smith 2000 [70]	ASD	RCT	Intervention group had significantly higher IQ [41],[42],[123] (development) (a) and lower levels of parent stress [124] (family) (d), but no differences were shown for language [48] (development) (a), daily skills [59] (b), or problem behavior [125] (c)	(a) range *d* = 0.37 to 0.76 (M = 0.54), (b) *d* = 0.11 (95% CI −0.64 to 0.85), (c) range *d* = 0.14 to 0.23 (M = 0.19), (d) *d* = 0.98 (95% CI −0.20 to 1.77)
Jocelyn 1998 [65]	ASD	RCT	Intervention group had significantly better language subscale scores (development) (a), but no differences in other developmental [126] (a), behavior [127] (b), or family outcomes [128],[129] (c)	(a) range *d* = −0.18 to 0.67 (M = 0.14), (b) range *d* = 0.20 to 0.41 (M = 0.31), (c) range *d* = −0.52 to 0.30 (M = −0.10)
Eikeseth 2012 [63]	ASD	QE	Intervention group had significantly higher scores in daily skills [59]	*d* = 0.93 (95% CI 0.38–1.47)
Peters-Scheffer 2010 [67]	ASD	QE	Intervention group had significantly higher IQ [38] (development) (a) and daily skills [59] (b), but no statistically significant difference in behavior [125] (c)	(a) *d* = 0.75 (95% CI 0.03–1.48), (b) *d* = 1.09 (95% CI 0.34–1.84), (c) *d* = 0.61 (95% CI −0.11 to 1.33)
Eikeseth 2007 [62],[72]	ASD	QE	Intervention group had significantly higher IQ [38],[45],[130] (development) (a) and daily skills [59] (b) at follow-up at age 8 y, and lower levels of aggression and socially inappropriate behavior [125] (behavior) (c)	(a) *d* = 0.56 (95% CI −0.25 to 1.38), (b) *d* = 1.20 (95% CI 0.33–2.07), (c) range *d* = 0.11 to 1.41 (M = 0.48)
Reed 2007 [68]	ASD	QE	Intervention group had a significantly better developmental outcome [131] (a) and daily skills [59] (b)	(a) range *d* = 0.34 to 0.47 (M = 0.41), (b) *d* = 0.54 (95% CI −0.19 to 1.27)
Remington 2007 [69],[73]	ASD	QE	Intervention group had significantly higher IQ [38],[41] (development) (a), improved outcomes on daily skills and motor subscales [59] (b), and fewer problem behaviors [132],[133] (c), but no differences for joint attention [48], (a), composite daily skills [59] (b), or family outcomes [134]–[136] (d)	(a) range *d* = 0.05 to 0.49 (M = 0.28), (b) *d* = 0.33 (95% CI −0.27 to 0.92), (c) *d* = 0.58 (95% CI: −0.07 to 1.23), (d) range *d* = −0.28 to 0.23 (M = −0.02)
Cohen 2006 [60]	ASD	QE	Intervention group had significantly higher scores for IQ and receptive language [38],[42] (development) (a) and daily skills [59] (b)	(a) range *d* = 0.41 to 0.61 (M = 0.48), (b) *d* = 0.69 (95% CI 0.07–1.31)
Howard 2005 [64]	ASD	QE	Intervention group had significantly higher scores for IQ [38],[40]–[47] and language [48]–[56] (development) (a) and daily skills [40],[59],[137],[138] (b)	(a) range *d* = 0.99 to 1.36 (M = 1.11), (b) *d* = 1.01 (95% CI 0.35–1.68)

No behavior analytic intervention studies were conducted in LMICs (LMIC status per World Bank).

ASD, autism spectrum disorder; *d*, Cohen's *d*; M, mean; QE, quasi-experimental study; RCT, randomized controlled trial.

**Table 7:** Research design, results, and effect sizes for cognitive rehabilitation, training, and support intervention studies.

First Author and Year of Publication of Original Study [Reference]	Child Diagnosis	Design	Results by Outcome	Effect Size by Outcome
Browder 2012 [76],[85]	ASD, ID	RCT	Intervention group had significantly better literacy scores [139] (school performance) (b), with smaller effects for the language outcome [49],[140] (development) (a)	(a) *d* = 0.30 (95% CI −0.11 to 0.71, (b) range *d* = 0.44 to 0.50 (M = 0.47)
Burgoyne 2012 [77]	ID	RCT	Intervention group showed superior performance on early reading skills [141],[142] (school performance) (b), but no difference in standardized language tests [55],[56] (development) (a)	(a) range *d* = 0.04 to 0.16 (M = 0.10), (b) range −0.25 to 0.71 (M = 0.25)
Allor 2010 [75],[84]	ASD, ID	RCT	Intervention group had significantly higher scores for blending nonwords, segmenting words, and word attack (school performance) (b), but no significant differences for development [49],[140] (a) and other school performance [143]–[145] (b)	(a) range *d* = 0.32 to 0.45 (M = 0.38), (b) range *d* = 0.23 to 0.65 (M = 0.42)
Elwan 2010 [78] a	ID	RCT	T2 group had significantly better developmental outcomes [146],[147] than T1, T3, and control groups	Range *d* = 1.61 to 3.51 (M = 2.51)
Panerai 2009 [81]	ASD	QE	Natural setting intervention group had significantly higher developmental [44] (a) and daily skills [59] (b) outcomes	(a) *d* = 1.22 (95% CI 0.33–2.12), (b) *d* = 0.87 (95% CI 0.00–1.73)
Goetz 2008 [79]	ID	QE	Intervention group had significantly higher scores on letter knowledge and early word recognition but not for word and nonword reading [115],[131],[148],[149] (school performance)	Range *d* = 0.29 to 1.57 (M = 0.93)
Perez 2008 [82]	ID	QE	Intervention group had significantly higher scores on mathematics, language arts, and social science examinations (school performance) (b), but minimal differences were found for IQ [41] (development) (a)	(a) *d* = 0.37 (95% CI −0.02 to 0.73), (b) range *d* = 1.39 to 1.61 (M = 1.47)
Tsang 2007 [83] a	ASD	QE	Both groups made gains, but intervention group had significantly lower scores on development [42],[150] (a) and daily skills [151] (b)	(a) range *d* = −1.27 to −0.98 (M = −1.13), (b) *d* = −1.89 (95% CI −2.70 to 1.08)
Jespen 2002 [80]	ASD, ID	QE	Intervention group showed modest gains between pre- and post-intervention for developmental outcomes [152] (a), daily skills [153] (b), and school performance [154] (c); differences between groups after intervention were not statistically significant	(a) *d* = 0.39 (95% CI −0.19 to 0.97), (b) range *d* = −0.70 to 0.73 (M = 0.25), (c) range *d* = 0.13 to 0.21 (M = 0.17)

^a^ LMIC status according to the World Bank.

ASD, autism spectrum disorder; *d*, Cohen's *d*; ID, intellectual disability; M, mean; QE, quasi-experimental study; RCT, randomized controlled trial; T1, T2, T3, treatment groups for studies with multiple treatments.

**Table 8:** Research design, results, and effect sizes for parent training intervention studies.

First Author and Year of Publication of Original Study [Reference]	Child Diagnosis	Design	Results by Outcome	Effect Size by Outcome
Wong 2010 [94] a	ASD	RCT	Intervention group showed significantly better symbolic play after treatment compared to control [155],[156] (development) and but no difference for parental stress [157] (family)	Unable to calculate effect size because data for groups were combined
Shin 2009 [91] a	ID	RCT	Both treatment and control groups showed gains across time in daily skills [59]; difference not significant	*d* = 0.09 (95% CI −0.63 to 0.80)
Plant 2007 [88]	ASD, ID	RCT	Both intervention groups had significantly fewer problem behaviors [158] (a) but no differences were found for maternal distress [159]–[162] (family) (b)	(a) range 0.33 to 0.99 (M = 0.61), (b) range *d* = −0.30 to 0.82) (M = 0.30)
Del Giudice 2006 [86]	ID	RCT	Intervention group had significantly better scores for the developmental outcome [39]	*d* = 1.47 (95% CI 0.65–2.28)
Roberts 2006 [89]	ASD, ID	RCT	Intervention group showed significant decreases in problem behavior [133] (a) and more appropriate parenting techniques [159],[161],[163] (family) (b)	(a) *d* = 0.69 (95% CI 0.15–1.24), (b) range *d* = 0.19 to 1.00 (M = 0.64)
Russell 1999 [90] a	ID	RCT	Intervention group had significantly better scores on all family outcomes except attitude towards intellectual disability, which was unchanged in both groups [164]	Range *d* = −0.13 to 1.01 (M = 0.61)
Varma 1992 [93] a	ID	RCT	Intervention group had significantly better developmental [165]–[168] (a), behavioral [169] (b), and family outcomes [164],[170],[171] (c)	(a) *d* = 0.63 (95% CI 0.18–1.08), (b) *d* = 0.54 (95% CI 0.10–1.09), (c) range *d* = 0.87 to 1.43 (M = 1.15)
McConachie 2005 [87]	ASD, ID	QE	Intervention group had a significantly greater vocabulary [58] (development) (a), but no differences for behavior [172] (b) or family outcomes [135],[173] (c)	(a) *d* = 0.55 (95% CI −0.01–1.11), (b) *d* = 0.00 (95% CI −0.55–0.55), (c) range *d* = −0.53 to 0.05 (M = −0.18)
Shu 2005 [92]	ASD	QE	Statistically significant difference between treatment and control group not found for family outcomes [174],[175]	Range *d* = −2.40 to 0.31 (M = −1.05)

^a^ LMIC status according to the World Bank.

*d*, Cohen's *d*; M, mean; QE, quasi-experimental study; RCT, randomized controlled trial.

**Figure 2:**
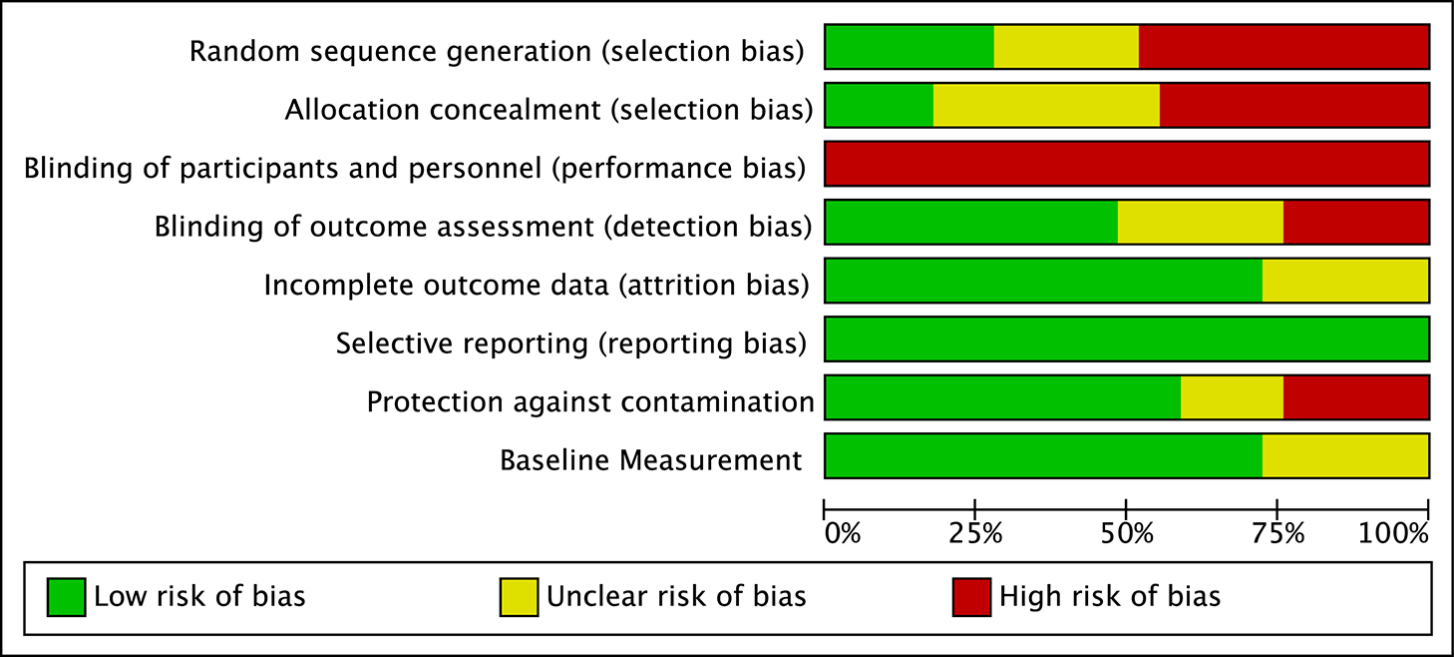
Risk of bias graph. Review authors' judgments about each risk of bias item, presented as percentages across all included studies.

Two independent raters assessed eight potential biases in all studies, which are shown as an average across all studies in Figure 2 and for each study by indicator in Figure S1. As seen in Figures 2 and S1, performance bias was a risk in all studies included in the review. This is likely due to the nature of psychosocial interventions, which involve interaction between providers and either the children with disabilities or the parents of the children. Given that many of the studies had low risk of detection bias, it is unclear what effect the high risk of performance bias might have had on the results. There was moderate risk of selection bias, most likely due to our inclusion of non-randomized studies. There was also moderate risk of contamination bias, which was due to the high risk of contamination in many of the behavior analytic studies that used eclectic comparison groups that potentially included elements of the behavioral treatments.

### Intervention Characteristics and Treatment Effects

**Figure 3:**
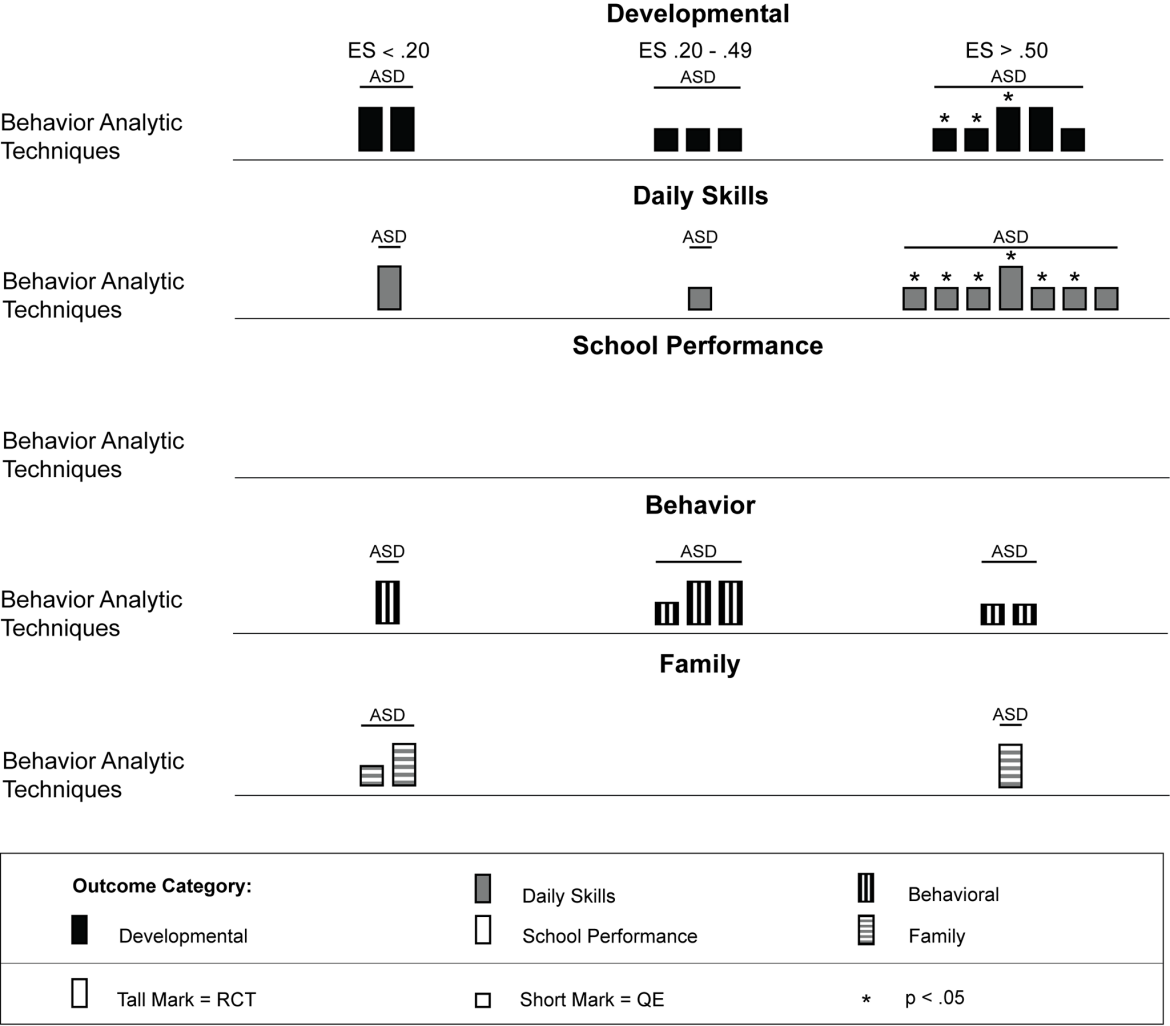
Harvest plot matrix of effect size estimates by outcome category for behavior analytic studies. ASD, autism spectrum disorders; ES, effect size; QE, quasi-experimental study; RCT, randomized controlled trial.

**Figure 4:**
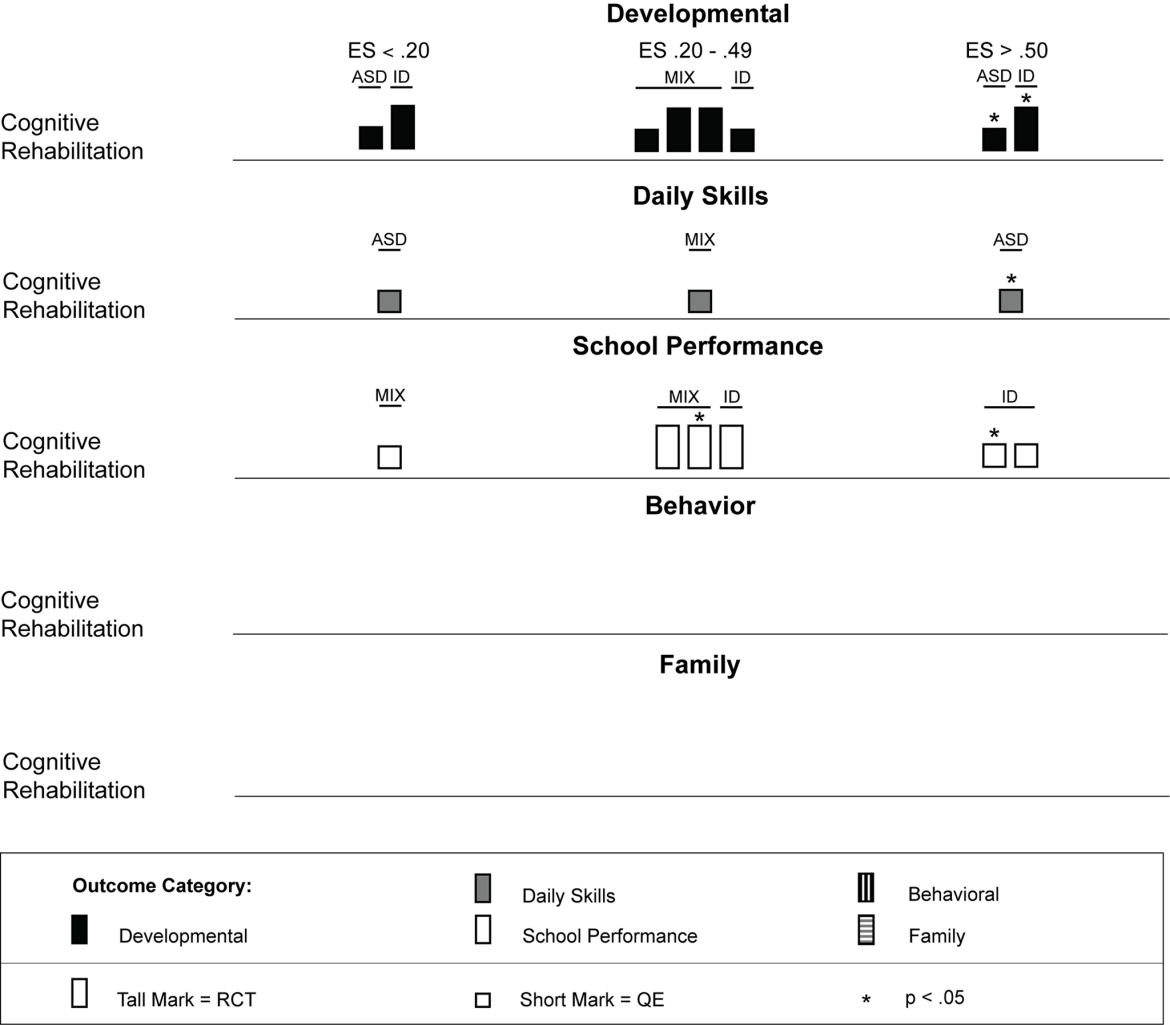
Harvest plot matrix of effect size estimates by outcome category for cognitive rehabilitation, training, and support studies. ASD, autism spectrum disorders; ES, effect size; ID, intellectual disability; MIX, autism spectrum disorders and intellectual disability; QE, quasi-experimental study; RCT, randomized controlled trial.

**Figure 5:**
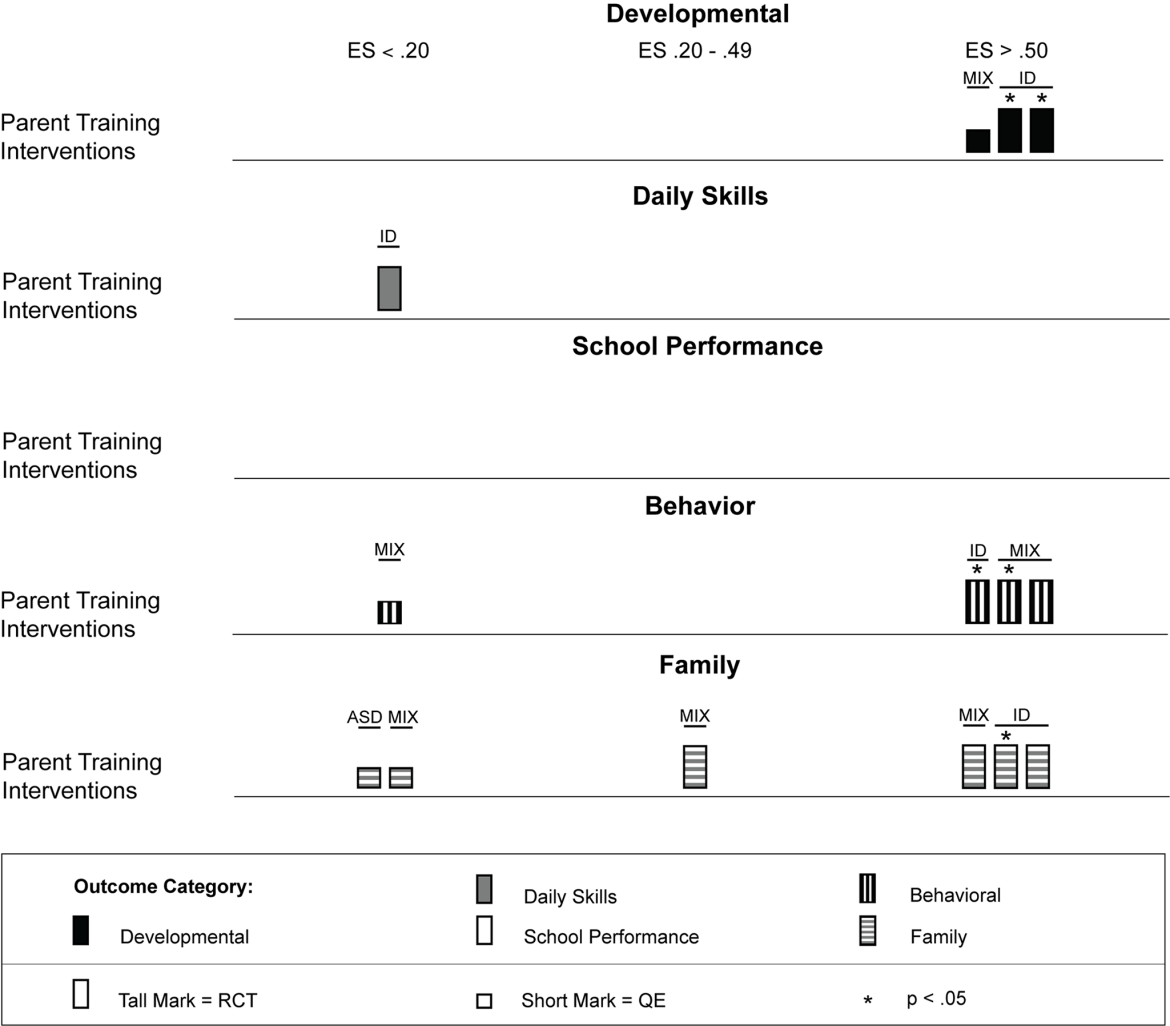
Harvest plot matrix of effect size estimates by outcome category for parent training interventions. ASD, autism spectrum disorders; ES, effect size; ID, intellectual disability; MIX, autism spectrum disorders and intellectual disability; QE, quasi-experimental study; RCT, randomized controlled trial.

Across studies there was a large range of effect size estimates, from a low of −1.89 to a high of 2.51. A large majority of the effect size estimates were positive (45 of 59, 76%), with just under half (29 of 59, 49%) being greater than 0.50, the threshold we took to suggest clinical significance. Eighteen of these 29 effect size estimates greater than 0.50 were statistically significant across intervention types. As shown in Figure 3, for the behavior analytic interventions, the best outcomes shown were for development and daily skills. For the studies of cognitive rehabilitation, training, and support, shown in Figure 4, the best outcomes were for developmental outcomes in children with intellectual disabilities between 6 and 11 y of age. For the parent training interventions, shown in Figure 5, the best outcomes were found for developmental, behavioral, and family outcomes. More detailed analyses, including subgroup analyses by type of intervention, are presented below.

#### Psychosocial interventions delivered using behavior analytic techniques

Fourteen articles representing 11 studies [60]–[73] described psychosocial interventions provided by non-specialist providers that used treatments based on the science of applied behavior analysis [74]. Table 2 provides descriptions of the intervention techniques and intervention density for each study, and Table 6 provides descriptions of the research characteristics and outcomes for each behavior analytic study. As shown in Table 6, four of 11 studies [61],[65],[66],[70] were randomized controlled trials, and zero studies were conducted in LMICs. As described in Table 1, these studies included 440 children with lower-functioning autism spectrum disorders who were, on average, under the age of 6 y at the onset of treatment. As shown in Figure 3, effect size estimates for the behavior analytic psychosocial interventions were generally robust, especially for daily skills, for which seven of nine (78%) effect size estimates were greater than 0.50, with six of seven effect size estimates greater than 0.50 having statistical significance. However, only one study with a large statistically significant effect size was a randomized controlled trial [61]. Mixed results were shown for developmental and behavioral outcomes, although two randomized controlled trials [61],[70] showed large effects for developmental outcomes, of which one was statistically significant. Only a few studies measured family outcomes, and no study of behavior analytic interventions examined school performance, even though three studies were conducted in a school setting [63],[66],[67].

**Figure 6:**
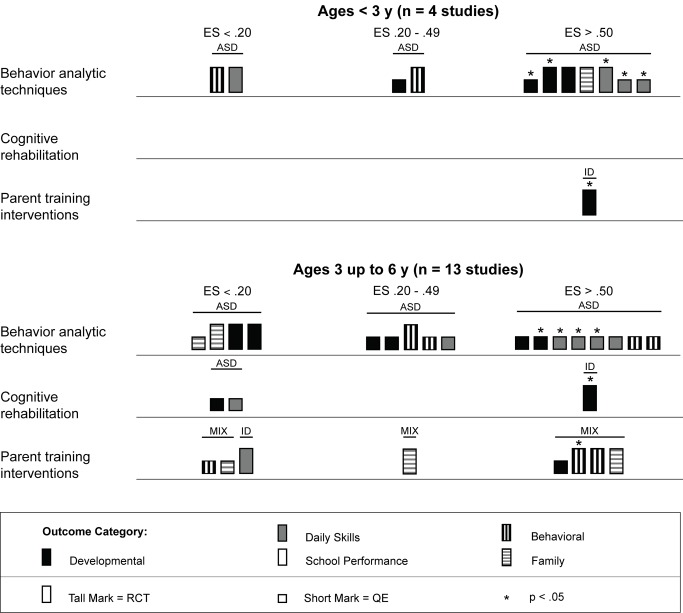
Harvest plot matrix comparison of effects by mean age of participants for children under 6 y old. ASD, autism spectrum disorders; ES, effect size; ID, intellectual disability; MIX, autism spectrum disorders and intellectual disability; QE, quasi-experimental study; RCT, randomized controlled trial.

**Figure 7:**
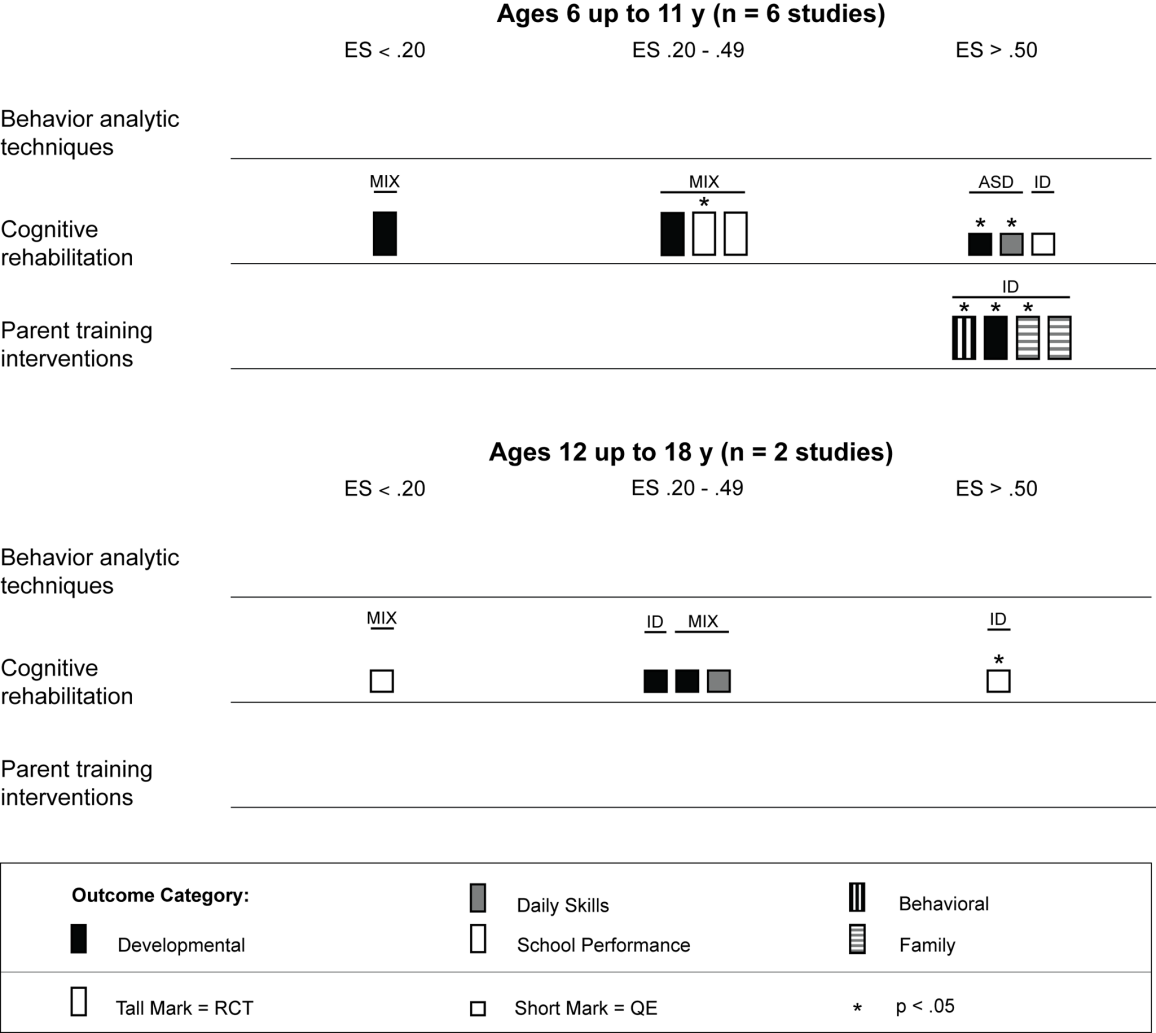
Harvest plot matrix comparison of effects by mean age of participants for children 6 y and older. ASD, autism spectrum disorders; ES, effect size; ID, intellectual disability; MIX, autism spectrum disorders and intellectual disability; QE, quasi-experimental study; RCT, randomized controlled trial.

The results from the comparison of mean participant age shown in Figure 6 suggests that behavior analytic techniques appear to be most effective for children under 3 y old, where four of seven effect size estimates >0.50 were found in randomized controlled trials [61],[70]. Five of seven (71%) effect size estimates for children under 3 y old were statistically significant, including two of four (50%) estimates from randomized controlled trials. Strong effects were also shown for children who were between 3 and 6 y old, although effect size estimates for three outcomes were <0.20. As shown in Figure 7, no behavior analytic studies included participants at pretreatment who were, on average, older than 6 y old.

**Figure 8:**
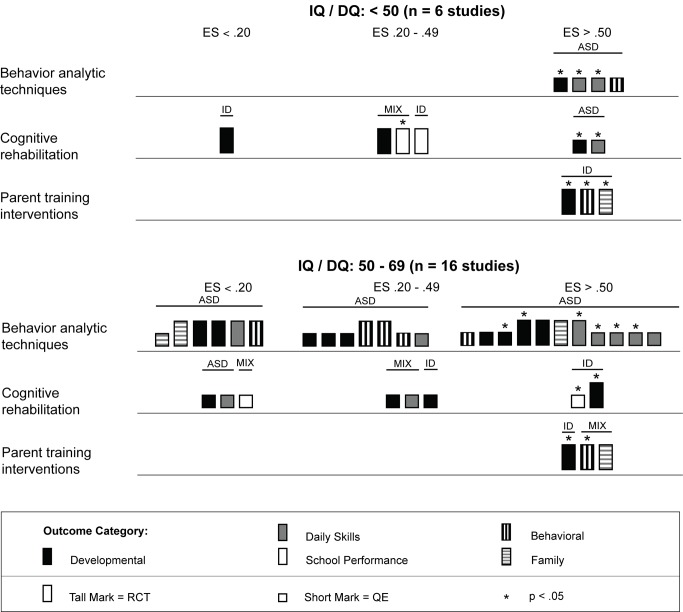
Harvest plot matrix comparison of effects by severity of intellectual disability. ASD, autism spectrum disorders; ES, effect size; ID, intellectual disability; MIX, autism spectrum disorders and intellectual disability; QE, quasi-experimental study; RCT, randomized controlled trial.

As shown in Figure 8, behavior analytic interventions seem quite effective for individuals with moderate to severe intellectual impairment; all effect size estimates from Eikeseth et al. [63] and Peters-Scheffer et al. [67] were greater than 0.50, with three of four (75%) estimates having statistical significance. For individuals with milder intellectual impairment, the behavior analytic interventions seemed effective at improving daily skills and developmental outcomes, which had, respectively, four and two effect size estimates greater than 0.50 that were statistically significant.

**Figure 9:**
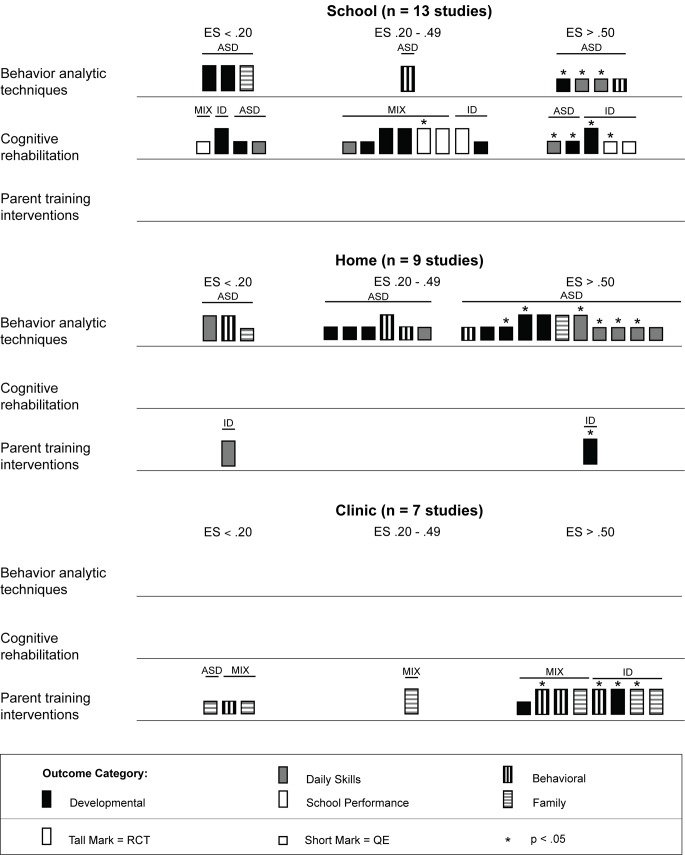
Harvest plot matrix comparison of effects by intervention setting. ASD, autism spectrum disorders; ES, effect size; ID, intellectual disability; MIX, autism spectrum disorders and intellectual disability; QE, quasi-experimental study; RCT, randomized controlled trial.

Figure 9 shows that behavior analytic interventions provided in schools had mixed effects; four of eight (50%) effect size estimates showed strong effects (three of four were statistically significant), and four of eight (50%) effect size estimates showed weak or no effects. For interventions conducted in a home setting, 11 of 20 (55%) effect size estimates were greater than 0.50, with six of the 11 (55%) estimates having statistical significance. In home and school settings, daily skills outcomes showed the most promising effects. Differential effects for other outcomes across settings showed inconclusive results (e.g., behavioral and family outcomes).

**Figure 10:**
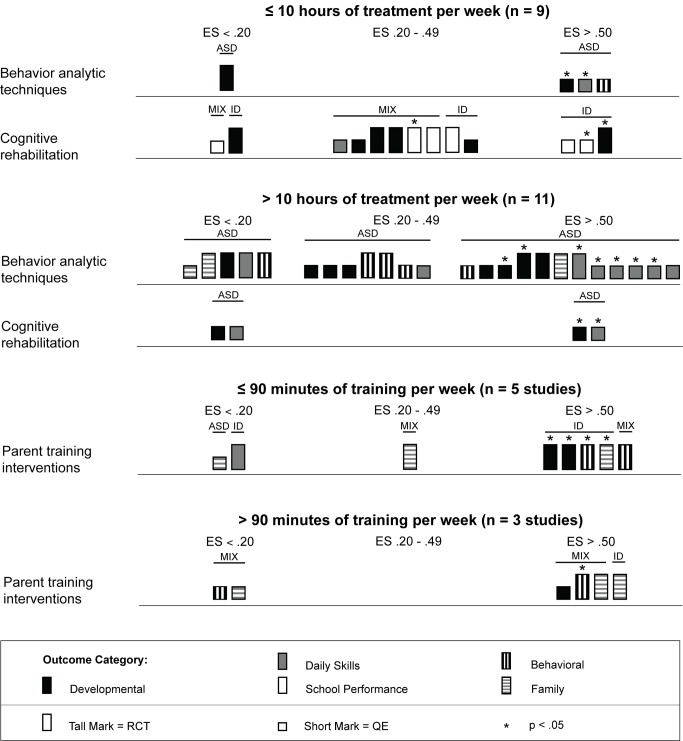
Harvest plot matrix comparison of effect by treatment density. ASD, autism spectrum disorders; ES, effect size; ID, intellectual disability; QE, quasi-experimental study; RCT, randomized controlled trial.

As shown in Table 2, the majority of behavior analytic studies involved intensive amounts of treatment; nine of 11 (82%) studies provided children with more than 10 h of treatment per week, with durations frequently lasting at least 52 wk. Many of these interventions also had significant amounts of treatment provider supervision, with some studies employing complex multilevel supervision arrangements with intensive training requirements [64],[70]. That the majority of behavior analytic studies involved intense amounts of treatment is also depicted in Figure 10, in which a majority of the effect size estimates in the plot are contained in the second panel (the panel showing studies with >10 h of treatment per week). However, the majority of these estimates came from studies that did not use random assignment to groups, and only seven of 12 (58%) effects were statistically significant. Additionally, there were no comparisons of intervention intensity in any of the studies included in this review; thus, it is not possible to conclude a true relation between treatment density and outcome. Overall, the effects for the high-intensity interventions were somewhat mixed, whereas the outcomes for the interventions with fewer than 10 h of treatment per week showed some promising outcomes. Jocelyn et al. [65] found significant improvement in standardized language scores, but not other developmental outcomes, using an intervention with moderate weekly density (mean = 21 h) with a shorter duration than most behavior analytic treatments (12 wk). Two studies examined interventions with densities lower than 10 h per week, with Kaale et al. [66] finding mixed results (significant increases in some but not all aspects of joint attention), and Peters-Scheffer et al. [67] finding strong effects for IQ, daily skills, and behavior, with effect sizes for all exceeding *d* = 0.60.

#### Psychosocial interventions delivered using cognitive rehabilitation, training, and support

We located 11 articles representing nine studies [75]–[85] involving 480 children with intellectual disabilities or lower-functioning autism spectrum disorders in which non-specialist providers delivered cognitive rehabilitation, training, and support interventions. Table 3 provides descriptions of the intervention techniques and intervention density for each study, and Table 7 provides descriptions of the research characteristics and outcomes for each cognitive rehabilitation, training, and support study. Four of nine (44%) studies [75]–[78] were randomized controlled trials, and two studies were conducted in LMICs [78],[83]. All studies occurred in school settings, with some focusing on specific curricular areas [75]–[77] and others incorporating curricular approaches to whole day instruction [81],[82]. As shown in Table 5, both teachers and aides, with different training and supervisory requirements across studies, delivered interventions. All outcomes addressed using cognitive rehabilitative strategies showed mixed effects, but, as illustrated in Figure 4, developmental and school performance outcomes showed the strongest effects (although only one of the four effect size estimates >0.50, which was statistically significant, was from a study that used a randomized controlled trial design [78]).

As shown across tables, the cognitive rehabilitation studies included participants older than 4 y with a mixture of diagnostic history with all levels of intellectual disability. Overall, the cognitive rehabilitation studies had the highest mean participant chronological ages; no studies had a mean age less than 3 y, and seven of nine (78%) studies had mean chronological ages older than 6 y. The cognitive rehabilitation category had the only studies with adolescents in this review [80],[82]. As shown in Figures 6 and 7, the results of the studies were mixed across age ranges, with no range showing superior effects over another. Likewise, as shown in Figure 8, the results were also mixed with respect to level of cognitive impairment. Given the small number of studies, compounded by the variability in intervention methods and intervention density, it is difficult to ascertain a true relation between cognitive ability and intervention success.

Intervention density was directly related to the overall approach, with focal content interventions having relatively low densities of about 2 h per week and whole day curricular approaches typically having densities in excess of 30 h per week. As shown in Figure 10, there is not clear evidence that greater intervention density was related to greater intervention effects, with 50% or fewer estimates for both density categories (≤10 h per week and >10 h per week) having effect size estimates greater than 0.50.

#### Psychosocial interventions delivered using parent training

We located nine studies [86]–[94] involving 368 children with intellectual disabilities or lower-functioning autism spectrum disorders in which the child's parents received a parent training intervention delivered by a non-specialist provider. Table 4 provides descriptions of the intervention techniques and intervention density for each study, and Table 8 provides descriptions of the research characteristics and outcomes for each parent training intervention study. Seven of nine studies were randomized controlled trials, and four studies, all randomized controlled trials, were conducted in LMICs [90],[91],[93],[94]. Most studies we located examined parent training interventions that were focused on teaching parents how to provide therapy services to their child; one study [92] focused mostly on improving parental well-being. As shown in Figure 5, the strongest effects were shown in developmental, behavioral, and family outcomes, with each outcome having three effect size estimates greater than 0.50, with many of these outcomes shown in randomized controlled trials [86],[88]–[90],[93], and with five of nine of the effect size estimates, all from randomized controlled trials, having statistical significance. As shown in Figures 6 and 7, the parent training interventions were most effective for parents of primary-school-aged children, where all four effect size estimates, three of which were statistically significant and all of which were from randomized controlled trials, were >0.50 [90],[93].

As described across tables and shown across figures, studies of parent training interventions typically included samples that were exclusively children with intellectual disability or studies that were a mixture of children with intellectual disability and lower-functioning autism spectrum disorders. The strongest effects were shown for individuals with intellectual disability without autism (five of six effect size estimates >0.50). Given the lack of specificity of child developmental level (five studies did not report a specific level; see Table 1 and Figure 8), we were unable to draw conclusions about whether parent training interventions were more or less effective for children with mild or more severe levels of intellectual impairment. Likewise, since seven of nine (78%) studies were conducted in clinical settings, comparison of clinic-based and home-based parent training programs was not possible (see Table 5 and Figure 9).

Compared to the behavior analytic interventions, the parent training interventions had much lower intervention densities, typically one or two 60- to 120-min sessions per week for 8 to 16 wk (see Table 4). As shown in Figure 10, five of eight (63%) effect size estimates, four of which were statistically significant, and three of six (50%) effect size estimates, one of which was statistically significant, were greater than 0.50 for the lower-density (≤90 min per week) and higher-density (>90 min per week) categories, respectively. All effect size estimates for the lower-density category came from randomized controlled trials, and three of four large effect size estimates for the higher-density category came from randomized controlled trials. Although it does not appear that increased density was systematically related to greater effects, it should be noted that these programs were often provided in conjunction with, not replacing, the child's typical school or early intervention programming, which complicates our ability to draw definitive conclusions.

## Discussion

### Summary of Main Findings

This review shows that there is a range of psychosocial interventions for individuals with intellectual disabilities or lower-functioning autism spectrum disorders that can be provided by non-specialist service providers. Overall, the outcomes of the studies included in this review show that non-specialist providers can deliver effective treatments to children with intellectual disabilities or lower-functioning autism spectrum disorders. As stated earlier, there was a large range of effect size estimates, from a low of −1.89 to a high of 2.51; a large majority of the effect size estimates were positive (45 of 59, 76%), with just under half (29 of 59, 49%) being greater than 0.50, likely indicating clinically significant effects. Eighteen of the 29 effect size estimates greater than 0.50 were statistically significant across intervention types. For the behavior analytic interventions, the best outcomes were shown for development and daily skills, especially for children with more severe levels of cognitive impairment at treatment onset. Cognitive rehabilitation, training, and support were found to be most effective for improving developmental outcomes in children with intellectual disabilities between 6 and 11 y of age, with mixed effects shown for daily skills and school performance. Finally, we found parent training interventions to be most effective for improving developmental, behavioral, and family outcomes when training was conducted in clinical settings. The strongest evidence from randomized controlled trials was found for parent training interventions, which had seven randomized controlled trials. For the parent training interventions, eight of nine effect size estimates greater than 0.50 were found for developmental, behavioral, and family outcomes, with five of the eight estimates from randomized controlled trials having statistical significance. Although our methods preclude a formal sensitivity analysis, examination of the tall marks in Figures 3–5 (which are representative of studies conducted using a randomized controlled trial design) shows the greatest amount of evidence for developmental and family outcomes for behavior analytic and parent training interventions, and robust findings for behavioral outcomes for parent training interventions. It is also noteworthy that the greatest percentage of studies with randomized controlled trials involved parent training interventions, where seven of nine (78%) studies were conducted using this design. Collectively, our review shows that beneficial effects can be realized when non-specialist providers deliver psychosocial interventions to children with intellectual disabilities or lower-functioning autism spectrum disorders.

### Relevance of Findings to Low-Resource Settings

The generation of evidence to inform practices in low-resource contexts, and specifically in LMICs, was part of the rationale for this review. We considered many factors during the scoping of the review to ensure that the results could be applied to LMICs, based on previous work by the World Health Organization's Department of Mental Health and Substance Abuse in developing evidence-based recommendations for LMICs [11],[95]. First, we only included studies that reported the outcomes of interventions delivered by non-specialist providers in community-based settings to increase the directness of evidence. All studies about interventions delivered by members of a research team or by specialists were excluded. In addition, by making explicit the information concerning a number of feasibility and contextual issues—including (a) the number of hours of training required to learn treatment techniques, (b) the requirements for supervision of treatment providers, (c) the intensity of interventions, and (d) the professional and education backgrounds of the people delivering the intervention—we allow readers to assess the applicability of the evidence about the various psychosocial interventions to the specific context.

Our findings that psychosocial interventions can be effective when delivered by non-specialist providers have much relevance for improving access to care for children and adolescents with intellectual disabilities or lower-functioning autism spectrum disorders who live in both HICs and LMICs, but they are especially useful in low-resource settings. These findings have the potential to facilitate an increase in access to psychosocial interventions for persons with developmental disorders by promoting task shifting and human resource development approaches. Within this context, the findings from our review that are likely to be most relevant are those from the studies that provided therapies with lower treatment density (e.g., psychosocial interventions requiring less than 10 h per week of direct therapy, or parent training interventions that met for 90 min or less per week). Two behavior analytic studies [66],[67] and nine cognitive rehabilitation, training, and support studies [75]–[80],[82] had treatment densities under 10 h per week (see Figure 10). Generally, the effects shown in these studies were weaker than our overall findings, the exception being the study by Peters-Scheffer et al. [67], which delivered 5 to 10 h of applied behavior analysis on top of standard care, and Elwan and el Din [78], Goetz et al. [79], and Perez and Beltran [82], which all showed very strong effects in development or school performance using inclusion and cognitive training, phonological awareness, and individualized instruction, respectively. Five parent training studies [86],[88],[91]–[93] had treatment densities of less than 90 min per week, with three of five studies showing strong effects [86],[88],[93]. It is also important to note that many of the studies providing high treatment densities involved significant supervision of the treatment providers that was often done by highly trained professionals (e.g., specialists such as psychiatrists and psychologists). The regularity, duration, and density of supervision were not reported in enough detail and with enough consistency for us to draw conclusions about the possible effects on outcome, but Reichow and Wolery [96] found supervisor training to have a significant relation to outcome in a review of early intensive behavioral intervention for young children with autism spectrum disorders. In addition to the training and supervision needs, it is also important to take into account that providing care to children with developmental disorders can be psychologically demanding, and appropriate support mechanisms for non-specialist care providers also need to be established. Uncovering these variables has important and practical implications regarding the feasibility of scaling up these interventions in low-resource settings. A recent review of the most promising procedures used to train paraprofessionals to work with individuals with autism spectrum disorders identified only a small number of studies, and reported the lack of clear training effects [16]. The review suggested that performance feedback can be a potentially effective and efficient means of on-the-job training that requires further research. E-health approaches(i.e., the transfer of health resources and health care by electronic means, including specifically the delivery of information and capacity building to health care providers through the Internet and telecommunications) have the potential to be instrumental in allowing appropriate training and supervision of non-specialist professionals even when resources and the availability of specialists are limited [97].

### Relation of Findings to Other Reviews

To our knowledge, this is the first review of psychosocial interventions delivered by non-specialist providers for children with intellectual disabilities or lower-functioning autism spectrum disorders. Therefore, direct comparisons with extant reviews are not possible. However, there have been reviews of psychosocial interventions for children with autism and other neurodevelopmental disorders delivered by specialist providers. Three relevant recent reviews [17]–[19] focused specifically on studies conducted in LMICs. They showed that a small number of studies have been conducted examining psychosocial interventions for children with intellectual disabilities or autism spectrum disorders in LMICs, with overall positive effects being shown for the interventions. An extant review on the effectiveness of interventions for child and adolescent mental health problems in primary care showed that there is some evidence that treatment by non-specialist primary health care and community staff is effective, although the number of studies included was limited [98]. Strong findings were shown in our review for behavior analytic techniques, which are supported by many recent reviews [28],[99],[100]. There have been fewer reviews of what we categorized as cognitive rehabilitation, training, and support; thus, placing our findings in the context of other reviews is difficult. Finally, as with our overall positive findings for parent training interventions, there are a number of reviews suggesting parents are able to learn the skills necessary to deliver therapies to their children and that the children show beneficial effects from these interventions [24],[25]. The findings of our review thus complement the findings of other reviews and have strong relevance for improving care for children with developmental disorders or lower-functioning autism spectrum disorders living in low-resource settings. Collectively, we feel that the findings of our review are well in line with the findings across reviews and that our focus on non-specialist providers allows our review to remain unique and one on which future reviews can build.

### Future Directions and Research

Although we located many studies, further research is needed to increase the knowledge of the effects of interventions for children with intellectual disabilities or lower-functioning autism spectrum disorders delivered by non-specialist providers to better inform strategies for service and human resource development. First, future studies should aim to use true experimental designs (randomized controlled trials) with high ecological validity to increase confidence in the effects of this class of interventions. Second, research looking at the effectiveness of capacity building strategies (including distance learning and e-health approaches for training and supervision) in improving non-specialists' ability to deliver psychosocial interventions, and at the quality of care received by children with developmental disorders, is needed. Third, research is needed on delivery strategies and resource requirements for providing psychosocial interventions for developmental disorders in low-resource settings as part of existing primary health care systems. Fourth, research on mediators and moderators of treatment effects is needed to identify the active and necessary components of treatment models [26]. Finally, research in all of these areas conducted specifically in LMICs will be most valuable in informing future care for persons with intellectual disabilities and lower-functioning autism spectrum disorders in areas with limited resources.

### Limitations

Although we took many steps, including protocol registry and use of the PRISMA checklist [20] (see Checklist S1), to ensure our review process limited potential sources of bias, no review is without limitation. One limiting factor of all systematic reviews is the quality of the included studies. The inclusion of non-randomized trials in and of itself introduces potential bias [31], and thus must be considered a limitation, although recent work has suggested non-randomized studies with high ecological validity can be an important and valid source of evidence [101]. The inclusion of both randomized and non-randomized trials and the variability of intervention techniques and outcomes precluded synthesizing studies statistically, which can also be seen as a limitation. Instead, we chose to present graphical depictions of effect using harvest plots, which highlighted that there was strong support from randomized controlled trials for parent training interventions to improve developmental, behavioral, and family outcomes. With respect to other risks of bias, as shown in Figures 2 and S1, no study was free of all risks of bias, which has potential impacts on the conclusions reached in this review. Of particular concern is the risk of performance bias, which, by the nature of psychosocial interventions, was high across all studies and must be considered a limitation and taken into consideration when interpreting the findings of this review. Another potential limitation is that most of the studies were conducted in North America or Europe, in HICs. Our stated purpose was to develop recommendations for LMICs, and the lack of ecological validity renders this difficult. It should be noted that almost half of the parent training studies were conducted using randomized controlled trials in LMICs, which might provide the most relevant findings from which to draw conclusions. It should also be noted that many of the non-specialist providers do in fact receive training, e.g., a teacher typically attends a college or university education program and often obtains licensure. However, many of these individuals might not have received the often extensive amounts of training to develop the skills to provide treatments to individuals with intellectual disabilities or lower-functioning autism spectrum disorders that specialist receive. We also cannot rule out the possibility of publication bias, as it was beyond the scope of this project to locate studies published in gray material. We tried to counter this with an extensive search across numerous global and localized databases. Finally, we limited the participant population to individuals who had an intellectual disability, which might limit the generalizability of our results to other populations of individuals with other developmental disorders, including those with higher-functioning autism spectrum disorders.

## Conclusion

The findings of this review support the delivery of psychosocial interventions by non-specialist providers to children who have intellectual disabilities or lower-functioning autism spectrum disorders. For the behavior analytic interventions, the best outcomes were shown for development and daily skills; cognitive rehabilitation, training, and support were found to be most effective for improving developmental outcomes, and parent training interventions to be most effective for improving developmental, behavioral, and family outcomes. We also conducted additional subgroup analyses using harvest plots. Given the scarcity of specialists in many low-resource settings, including many LMICs, these findings may provide guidance for scale-up efforts for improving outcomes for children with developmental disorders or lower-functioning autism spectrum disorders.

## Supporting Information

Checklist S1 **PRISMA checklist** [**20**] **.**
(DOC)

Figure S1 **Risk of bias summary.** Review authors' judgments about each risk of bias item for each included study, where green indicates low risk of bias, yellow indicates unclear risk of bias, and red indicates high risk of bias.
(TIF)

Table S1 **Outcome measures and outcome categories for included studies.**
(DOCX)

Text S1 **Sample search strategy for Medline (1946 to week 2 of June 2013), Cumulative Index to Nursing and Allied Health (1981 to 24 June 2013), and Embase (1974 to 24 June 2013).**
(DOCX)

Text S2 **Search strategy for PsycINFO (1967 to week 3 of June 2012).**
(DOCX)

Text S3 **Sample search strategies for African Index Medicus, AFRO Library, and Western Pacific Region Index Medicus.**
(DOCX)

Text S4 **Search strategy for Literatura Latino-Americana e do Caribe em Ciências da Saúde.**
(DOCX)

Text S5 **Search strategy for Cochrane Central Register of Controlled Trials (24 June 2013).**
(DOCX)

Text S6 **List of excluded studies, with reason.**
(DOCX)

## Abbreviations

HIC: higher-income country
DQ: developmental quotient
LMIC: lower- and middle-income country
PRISMA: Preferred Reporting Items for Systematic Reviews and Meta-Analyses
