# Supplementary figures and images for: Non-Specialist Psychosocial Interventions for Children and Adolescents with Intellectual Disability or Lower-Functioning Autism Spectrum Disorders: A Systematic Review
Source: PLoS Med. 2013 Dec 17;10(12):e1001572. doi: 10.1371/journal.pmed.1001572 (PMC3866092; doi:10.1371/journal.pmed.1001572)

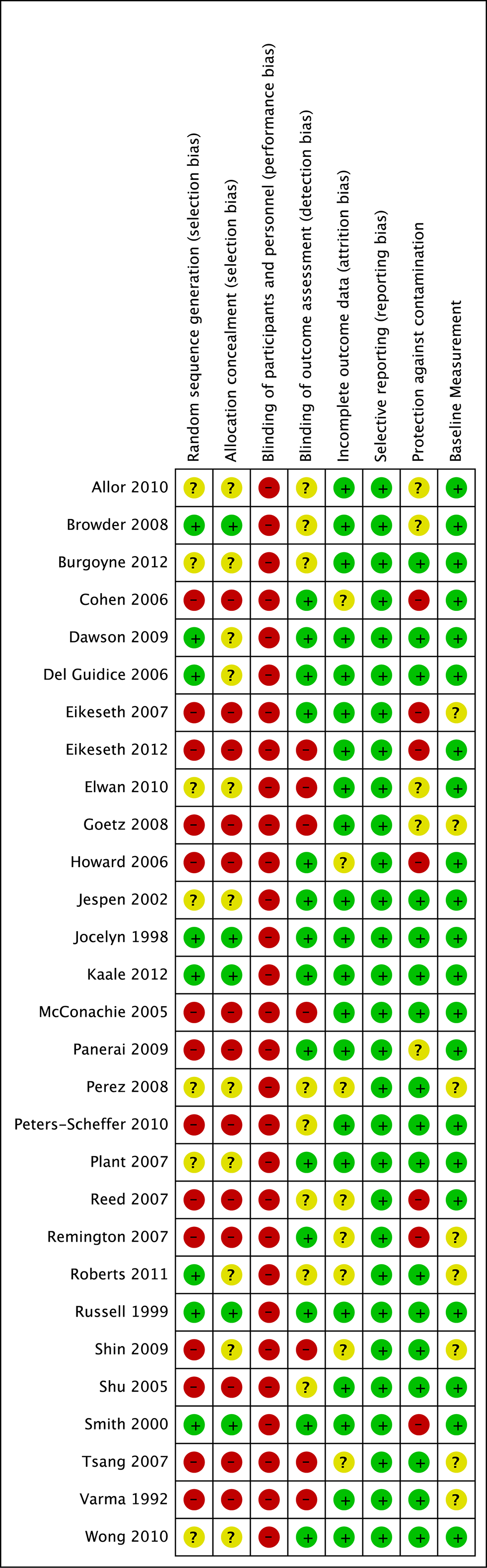

Supplement: Figure S1 — Risk of bias summary. Review authors' judgments about each risk of bias item for each included study, where green indicates low risk of bias, yellow indicates unclear risk of bias, and red indicates high risk of bias. (TIF) [file pmed.1001572.s002.tif]
