# Supplementary material for: Non-Specialist Psychosocial Interventions for Children and Adolescents with Intellectual Disability or Lower-Functioning Autism Spectrum Disorders: A Systematic Review
Source: PLoS Med. 2013 Dec 17;10(12):e1001572. doi: 10.1371/journal.pmed.1001572 (PMC3866092; doi:10.1371/journal.pmed.1001572)
Supplement: Table S1 — Outcome measures and outcome categories for included studies. (DOCX) [file pmed.1001572.s003.docx]

**Table S1. Outcome Measures and Outcome Categories for Included Studies**

| Study | Outcome Measures and Outcome Category |
| --- | --- |
| **Behavior Analytic Techniques** | |
| Cohen 2006 [[60](#_ENREF_60)] | Standardized IQ tests [[38](#_ENREF_38),[42](#_ENREF_42)], Reynell Developmental Language Scales-3^rd^ Ed. [[48](#_ENREF_48)] (developmental, a), Vineland Adaptive Behavior Scales [[59](#_ENREF_59)] (daily skills, b) |
| Dawson 2010 [[61](#_ENREF_61),[71](#_ENREF_71)] | Mullen Scales of Early Learning [[37](#_ENREF_37)] (developmental, a), Vineland Adaptive Behavior Scales [[59](#_ENREF_59)] (daily skills, b), Repetitive behavior scale [[122](#_ENREF_122)] (behavior, c) |
| Eikeseth 2007 [[62](#_ENREF_62),[72](#_ENREF_72)] | Standardized IQ tests [[38](#_ENREF_38),[45](#_ENREF_45),[130](#_ENREF_130)] (developmental, a), Vineland Adaptive Behavior Scales [[59](#_ENREF_59)] (daily skills, b), Child Behavior Checklist [[125](#_ENREF_125)] (behavior, c) |
| Eikeseth 2012 [[63](#_ENREF_63)] | Vineland Adaptive Behavior Scales [[59](#_ENREF_59)] (daily skills) |
| Howard 2005 [[64](#_ENREF_64)] | Standardized IQ tests [[38](#_ENREF_38),[40-47](#_ENREF_40)], Standardized language tests [[48-56](#_ENREF_48)] (developmental, a), Standardized adaptive behavior scales [[40](#_ENREF_40),[59](#_ENREF_59),[137](#_ENREF_137),[138](#_ENREF_138)] (daily skills, b) |
| Jocelyn 1998 [[65](#_ENREF_65)] | Early Intervention Developmental Profile [[126](#_ENREF_126)] (developmental, a), Autism Behavior Checklist [[127](#_ENREF_127)] (behavior, b), Stress-Arousal Checklist [[129](#_ENREF_129)], Family Assessment Measure [[128](#_ENREF_128)] (family, c) |
| Kaale 2012 [[66](#_ENREF_66)] | Early Social Communication Scale [[57](#_ENREF_57)] (developmental) |
| Peters-Scheffer 2010 [[67](#_ENREF_67)] | Bayley Scales of Infant Development [[38](#_ENREF_38)] (developmental, a), Vineland Adaptive Behavior Scales [[59](#_ENREF_59)] (daily skills, b), Child Behavior Checklist [[125](#_ENREF_125)] (behavior, c) |
| Reed 2007 [[68](#_ENREF_68)] | British Ability Scale-II [[131](#_ENREF_131)], Psychoeducational Profile-Revised [[44](#_ENREF_44)] (developmental, a), Vineland Adaptive Behavior Scales [[59](#_ENREF_59)] (daily skills, b) |
| Remington 2007 [[69](#_ENREF_69),[73](#_ENREF_73)] | Standardized IQ tests [[38](#_ENREF_38),[41](#_ENREF_41)], Standardized language tests [[48](#_ENREF_48),[57](#_ENREF_57)] (developmental, a), Vineland Adaptive Behavior Scales [[59](#_ENREF_59)] (daily skills, b), Developmental Behavior Checklist [[132](#_ENREF_132),[133](#_ENREF_133)] (behavior, c), Hospital Anxiety and Depression Scale [[134](#_ENREF_134)], Questionnaire on Resources and Stress-Friedrich Short Form [[135](#_ENREF_135)] Kansas Inventory of Parental Perceptions [[136](#_ENREF_136)] (family, d) |
| Smith 2000 [[70](#_ENREF_70)] | Standardized IQ tests [[41](#_ENREF_41),[42](#_ENREF_42),[123](#_ENREF_123)], Reynell Developmental Language Scales-3^rd^ Ed. [[48](#_ENREF_48)] (developmental, a), Vineland Adaptive Behavior Scales [[59](#_ENREF_59)] (daily skills, b), Child Behavior Checklist [[125](#_ENREF_125)] (behavior, c), Family Satisfaction Questionnaire [[124](#_ENREF_124)] (family, d) |
| **Cognitive Rehabilitation, Training, and Support** | |
| Allor 2010 [[75](#_ENREF_75),[84](#_ENREF_84)] | Standardized language tests [[49](#_ENREF_49),[140](#_ENREF_140)], (developmental, a), Woodcock Language Proficiency Battery-Revised [[143](#_ENREF_143)], Comprehensive Test of Phonological Processing [[144](#_ENREF_144)], Test of Word Reading Efficiency [[145](#_ENREF_145)] (school performance, b) |
| Browder 2012 [[76](#_ENREF_76),[85](#_ENREF_85)] | Standardized language tests [[49](#_ENREF_49),[140](#_ENREF_140)], (developmental, a), Nonverbal Literacy Assessment [[139](#_ENREF_139)], (school performance, b) |
| Burgoyne 2012 [[77](#_ENREF_77)] | Standardized language tests [[55](#_ENREF_55),[56](#_ENREF_56)], (developmental, a), York Assessment of Early Reading Battery [[141](#_ENREF_141)], Action Picture Test [[142](#_ENREF_142)], (school performance, b) |
| Elwan 2010 [[78](#_ENREF_78)] * | Subtests from Kaufman Assessment Battery of Children [[146](#_ENREF_146)] and McCarthy Scales of Children’s Abilities [[147](#_ENREF_147)] (developmental) |
| Goetz 2008 [[79](#_ENREF_79)] | Letter knowledge, early word recognition, word reading [[115](#_ENREF_115),[148](#_ENREF_148),[149](#_ENREF_149)] and non word reading subscales of British Achievement Scales [[131](#_ENREF_131)] (school performance) |
| Jespen 2002 [[80](#_ENREF_80)] | Cognitive Assessment System [[152](#_ENREF_152)] (developmental, a), Adaptive Behavior Scale [[153](#_ENREF_153)] (daily skills, b), Woodcock-Johnson-Revised [[154](#_ENREF_154)] (school performance, c) |
| Panerai 2009 [[81](#_ENREF_81)] | Psychoeducational Profile-Revised [[44](#_ENREF_44)] (developmental, a), Vineland Adaptive Behavior Scales [[59](#_ENREF_59)] (daily skills, b) |
| Perez 2008 [[82](#_ENREF_82)] | Stanford-Binet (4^th^ ed) [[41](#_ENREF_41)] (developmental, a), mathematics, language, and social science examinations (school performance, b) |
| Tsang 2007 [[83](#_ENREF_83)] * | Merrill-Palmer [[42](#_ENREF_42)], Chinese Psychoeducational Profile-Revised [[150](#_ENREF_150)] (developmental, a), Hong Kong Based Adaptive Behavior Scales [[151](#_ENREF_151)] (daily skills, b) |
| **Parent Training Interventions** | |
| Del Giudice 2006 [[86](#_ENREF_86)] | Brunet-Lezine Psychomotor Development Scale [[39](#_ENREF_39)] (developmental) |
| McConachie 2005 [[87](#_ENREF_87)] | MacArthur Communicative Development Inventories [[58](#_ENREF_58)] (developmental, a), Behavior Screening Questionnaire [[172](#_ENREF_172)] (behavioral, b) Questionnaire on Resources and Stress [[135](#_ENREF_135)], Parent Feelings Questionnaire [[173](#_ENREF_173)] (family, c) |
| Plant 2007 [[88](#_ENREF_88)] | Eyberg Child Behavior Inventory, Developmental Behavior Checklist [[158](#_ENREF_158)] (behavior, a), Parenting Scale [[159](#_ENREF_159)], Parenting Sense of Competence Scale [[160](#_ENREF_160)], Depression, Anxiety, and Stress Scales [[161](#_ENREF_161)], Abbreviated Dyadic Adjustment Scale [[162](#_ENREF_162)] (family, b) |
| Roberts 2006 [[89](#_ENREF_89)] | Developmental Behavior Checklist [[133](#_ENREF_133)] (behavior, a), Family Observation Schedule-Revised III [[163](#_ENREF_163)], Parenting Scale [[159](#_ENREF_159)], Depression, Anxiety, & Stress Scale [[161](#_ENREF_161)] (family, b) |
| Russell 1999 [[90](#_ENREF_90)] * | Parental Attitude Scale Towards Management of Intellectual Disability [[164](#_ENREF_164)] (family) |
| Shin 2009 [[91](#_ENREF_91)] * | Vineland Adaptive Behavior Scales [[59](#_ENREF_59)] (daily skills) |
| Shu 2005 [[92](#_ENREF_92)] | Chinese Health Questionnaire-30 [[174](#_ENREF_174)], World Health Organization Quality of Life – short form [[175](#_ENREF_175)] (family) |
| Varma 1992 [[93](#_ENREF_93)] * | Multiple intelligence subtests [[165-168](#_ENREF_165)] (developmental, a), Bhat’s behavior rating scale [[169](#_ENREF_169)] (behavior, b), Marital adjustment [[170](#_ENREF_170)], parental attitudes [[164](#_ENREF_164)], and social burden [[171](#_ENREF_171)] (family, c) |
| Wong 2010 [[94](#_ENREF_94)] * | Symbolic Play Test [[155](#_ENREF_155)], Ritvo-Freeman Real Life Rating Scale [[156](#_ENREF_156)] (developmental), Parenting Stress Index Short Form [[157](#_ENREF_157)] (family) |
