# Supplementary material for: Non-Specialist Psychosocial Interventions for Children and Adolescents with Intellectual Disability or Lower-Functioning Autism Spectrum Disorders: A Systematic Review
Source: PLoS Med. 2013 Dec 17;10(12):e1001572. doi: 10.1371/journal.pmed.1001572 (PMC3866092; doi:10.1371/journal.pmed.1001572)
Supplement: Text S1 — Sample search strategy for Medline (1946 to week 2 of June 2013), Cumulative Index to Nursing and Allied Health (1981 to 24 June 2013), and Embase (1974 to 24 June 2013). (DOCX) [file pmed.1001572.s004.docx]

Text S1. Sample Search Strategy for MEDLINE (1946 to June Week 2 2013), Cumulative Index for Nursing and Allied Health (CINAHL; 1981 to 24 June 2013), and EMBASE (1988 to 24 June 2013)

1. exp child development disorders, pervasive/
2. exp Intellectual Disability/
3. rett.tw.
4. autis*.tw.
5. (pervasive development* disorder* or PDD or PDDs).tw.
6. (developmental adj delay*).tw.
7. (developmental adj disorder*).tw.
8. (developmental adj disability).tw.
9. (developmental adj disabilities).tw.
10. (developmental adj differen*).tw.
11. ((down* adj syndrome) or (fragile adj X)).tw.
12. (mental adj retard*).tw.
13. (intellectual adj disorder*).tw.
14. (intellectual adj disability).tw.
15. (intellectual adj disabilities).tw.
16. 1 or 2 or 3 or 4 or 5 or 6 or 7 or 8 or 9 or 10 or 11 or 12 or 13 or 14 or 15
17. behavior therapy/
18. exp treatment outcome/
19. (educat* or train* or program* or therap* or intervention* or treatment*).tw.
20. psychosocial.tw.
21. 17 or 18 or 19 or 20
22. 16 and 21
23. limit 22 to (yr=”1992 –Current” and “all child (0 to 18 years)”)
24. qualitative.mp.
25. 23 not 24
26. exp Epidemiologic Methods/
27. 25 and 26
