# Supplementary material for: Non-Specialist Psychosocial Interventions for Children and Adolescents with Intellectual Disability or Lower-Functioning Autism Spectrum Disorders: A Systematic Review
Source: PLoS Med. 2013 Dec 17;10(12):e1001572. doi: 10.1371/journal.pmed.1001572 (PMC3866092; doi:10.1371/journal.pmed.1001572)
Supplement: Text S2 — Search strategy for PsycINFO (1967 to week 3 of June 2012). (DOCX) [file pmed.1001572.s005.docx]

Text S2: Search Strategy for PsycINFO (1967 to June Week 3 2013)

1. exp pervasive developmental disorders/
2. (pervasive development* disorder* or PDD or PDDs).tw.
3. rett.tw.
4. autis*.tw.
5. (mental adj retard*).tw.
6. (intellectual adj disorder*).tw.
7. (intellectual adj disability).tw.
8. (intellectual adj disabilities).tw.
9. (developmental adj disorder*).tw.
10. (developmental adj delay*).tw.
11. (developmental adj disability).tw.
12. (developmental adj disabilities).tw.
13. (developmental adj differen*).tw.
14. ((down* adj syndrome) or (fragile adj X)).tw.
15. 1 or 2 or 3 or 4 or 5 or 6 or 7 or 8 or 9 or 10 or 11 or 12 or 13 or 14
16. behavior therapy/
17. exp treatment outcome/
18. (educat* or train* or program* of therap* or intervention* or treatment*).tw.
19. psychosocial.tw.
20. 16 or 17 or 18 or 19
21. 15 and 20
22. limit 21 to (1800 quantitative study and (100 childhood <birth to age 12 yrs> or 200 adolescence <age 13 to 17 yrs>) and yr=”1992 – Current”)
