# Supplementary material for: Non-Specialist Psychosocial Interventions for Children and Adolescents with Intellectual Disability or Lower-Functioning Autism Spectrum Disorders: A Systematic Review
Source: PLoS Med. 2013 Dec 17;10(12):e1001572. doi: 10.1371/journal.pmed.1001572 (PMC3866092; doi:10.1371/journal.pmed.1001572)
Supplement: Text S3 — Sample search strategies for African Index Medicus, AFRO Library, and Western Pacific Region Index Medicus. (DOCX) [file pmed.1001572.s006.docx]

Text S3. Sample Search Strategies for African Index Medicus, Afro Library (AFROLIB), and Index Medicus for the Western Pacific (WPRIM)

1. autism
2. autistic
3. autism spectrum disorder
4. ASD
5. pervasive developmental disorder
6. PDD
7. intellectual disability
8. developmental disability
9. developmental disorder
10. mental retardation
11. autistique
12. troubles du spectre autistique
13. troubles envahissants du developpement
14. PDD deficience intellectuelle
15. troubles du developpement
16. retard mental
17. 1 or 2 or 3 or 4 or 5 or 6 or 7 or 8 or 9 or 10 or 11 or 12 or 13 or 14 or 15 or 16
