# Supplementary material for: Non-Specialist Psychosocial Interventions for Children and Adolescents with Intellectual Disability or Lower-Functioning Autism Spectrum Disorders: A Systematic Review
Source: PLoS Med. 2013 Dec 17;10(12):e1001572. doi: 10.1371/journal.pmed.1001572 (PMC3866092; doi:10.1371/journal.pmed.1001572)
Supplement: Text S4 — Search strategy for Literatura Latino-Americana e do Caribe em Ciências da Saúde. (DOCX) [file pmed.1001572.s007.docx]

Text S4. Search strategy for Literatura Latino Americana em Ciências da Saúde (LILACS):

1. autism
2. autistic
3. autism spectrum disorder
4. ASD
5. pervasive developmental disorder
6. PDD
7. intellectual disability
8. developmental disability
9. developmental disorder
10. mental retardation
11. autismo
12. autista
13. trastorno del desarrollo
14. discapacidad intelectual
15. discapacidad del desarrollo
16. retraso mental
17. 1 or 2 or 3 or 4 or 5 or 6 or 7 or 8 or 9 or 10 or 11 or 12 or 13 or 14 or 15 or 16
