# Supplementary material for: Non-Specialist Psychosocial Interventions for Children and Adolescents with Intellectual Disability or Lower-Functioning Autism Spectrum Disorders: A Systematic Review
Source: PLoS Med. 2013 Dec 17;10(12):e1001572. doi: 10.1371/journal.pmed.1001572 (PMC3866092; doi:10.1371/journal.pmed.1001572)
Supplement: Text S5 — Search strategy for Cochrane Central Register of Controlled Trials (24 June 2013). (DOCX) [file pmed.1001572.s008.docx]

Text S5. Search strategy for Cochrane Central Register of Controlled Trials (CENTRAL; 24 June 2013)

1. autis*
2. pervasive development* disorder*
3. PDD
4. intellectual disability
5. intellectual disabilities
6. intellectual disorder*
7. mental retardation
8. developmental disability
9. developmental disabilities
10. developmental disorder*
11. 1 or 2 or 3 or 4 or 5 or 6 or 7 or 8 or 9 or 10

limit 11to year 1992 to 2012
