# Supplementary material for: Non-Specialist Psychosocial Interventions for Children and Adolescents with Intellectual Disability or Lower-Functioning Autism Spectrum Disorders: A Systematic Review
Source: PLoS Med. 2013 Dec 17;10(12):e1001572. doi: 10.1371/journal.pmed.1001572 (PMC3866092; doi:10.1371/journal.pmed.1001572)
Supplement: Text S6 — List of excluded studies, with reason. (DOCX) [file pmed.1001572.s009.docx]

**Text S6: List of Excluded Studies, with Reason**

Reasons for exclusion, with reference number from reference list below for each excluded study:

1. Did not meet participant criteria (n = 52).

Studies: 1, 4, 8, 26, 32, 34, 38, 40, 41, 46, 53, 58, 60, 61, 66, 70, 71, 73, 92, 97, 103, 107, 110, 111, 112, 121, 125, 128, 129, 137, 142, 143, 146, 147, 149, 150, 157, 168, 170, 171, 172, 174, 177, 178, 180, 183, 184, 192, 193, 194, 195, 199

2. Did not have at least two groups in which at least one group received treatment that was compared to another group who either did not receive treatment or received a different treatment (n = 63).

Studies: 5, 6, 7, 10, 11, 12, 13, 16, 18, 20, 21, 22, 27, 31, 33, 35, 48, 49, 52, 56, 57, 59, 65, 72, 78, 82, 84, 86, 87, 88, 89, 93, 94, 98, 100, 101, 102, 105, 108, 109, 113, 114, 117, 122, 123, 127, 136, 139, 140, 141, 148, 153, 158, 161, 162, 165, 173, 176, 181, 182, 185, 187, 188

3. Study was not prospective (n = 14).

Studies: 17, 43, 44, 45, 51, 55, 95, 99, 135, 138, 156, 167, 191, 200

4. Treatment was not psychosocial (n = 14).

Studies: 24, 37, 39, 62, 64, 74, 75, 77, 83, 85, 104, 120, 154, 166

5. Treatment or parent training was not delivered by a non-specialist (n = 54).

Studies: 2, 3, 9, 19, 23, 25, 28, 29, 30, 36, 42, 47, 50, 54, 63, 67, 68, 69, 76, 79, 80, 81, 90, 91, 96, 106, 115, 116, 118, 119, 124, 126, 130, 131, 132, 133, 134, 145, 151, 152, 155, 159, 160, 163, 164, 169, 175, 179, 186, 189, 190, 196, 197, 198

6. Could not locate article (n = 3).

Studies: 14, 15, 144

References:

1. Abrahamse ME, Junger M, Chavannes EL, Coelman FJG, Boer F, et al. (2012) Parent-child interaction therapy for preschool children with disruptive behaviour problems in the Netherlands. Child Adol Psychiatry Mental Health 6: 24.

2. Aldred C, Green J, Adams C (2004) A new social communication intervention for children with autism: Pilot randomized controlled treatment study suggesting effectiveness. J Child Psychol Psychiatry 45: 1420-1430.

3. Aldred C, Green J, Emsley R, McConachie H (2012) Mediation of treatment effect in a communication intervention for pre-school children with autism. J Autism Dev Disord 42: 447-454.

4. Alfassi M, Weiss I, Lifshitz H (2009) The efficacy of reciprocal teaching in fostering the reading literacy of students with intellectual disabilities. Eur J Spec Needs Educ 24: 291-305.

5. Anan RM, Warner LJ, McGillivary JE, Chong IM, Hines SJ (2008) Group Intensive Family Training (GIFT) for preschoolers with autism spectrum disorders. Behav Interv 23: 165-180.

6. Aparicio MTS, Balana JM (2003) Social Early Stimulation of Trisomy-21 Babies. Early Child Develop Care 173: 557-561.

7. Bachman JA, Bachman WG, Franzel AS, Marcus MC (1994) Preteaching developmentally delayed preschoolers to aid vision screening. Optom Vis Sci 71: 713-716.

8. Baghdadli A, Brisot-Dubois J, Picot MC, Michelon C (2010) [Comparison of the effect of two prosocial interventions about the evolution of recognition of facial expression abilities and social cognition of children with an Asperger syndrome or high functioning autism] TO: Comparaison de l'effet de deux interventions prosociales sur l'evolution des capacites d'identification des expressions faciales et du raisonnement social d'enfants avec un syndrome d'Asperger ou autisme de haut niveau LA: Fre. Neuropsychiatr Enfance Adol 58: 456-462.

9. Bagner DM, Eyberg SM (2007) Parent-child interaction therapy for discruptive behavior in children with mental retardation: A randomized controlled trial. J Clin Child Adolesc Psychol 36: 418-429.

10. Baharav E, Reiser C (2010) Using telepractice in parent training in early autism. Telemed and E Health 16: 727-731.

11. Baker-Ericzen MJ, Stahmer AC, Burns A (2007) Child Demographics Associated With Outcomes in a Community-Based Pivotal Response Training Program. J Posit Behav Interv 9: 52-60.

12. Baltruschat L, Hasselhorn M, Tarbox J, Dixon DR, Najdowski AC, et al. (2011) Addressing working memory in children with autism through behavioral intervention. Res Autism Spectr Disord 5: 267-276.

13. Baltruschat L, Hasselhorn M, Tarbox J, Dixon DR, Najdowski AC, et al. (2011) Further analysis of the effects of positive reinforcement on working memory in children with autism. Res Autism Spectr Disord 5: 855-863.

14. Banerjee M (2006) Autism & communication-A psycho-bio-social approach. Indian J Comm Psychol 2: 1-15.

15. Banerjee M, Ray P, Panda A (2013) Role of sex education on odd sexual and problem behaviour: A study on adolescents with autism. Indian J of Comm Psychol 9: 1-20.

16. Basil C, Reyes S (2003) Acquisition of literacy skills by children with severe disability. Child Lang Teach Ther 19: 27-48.

17. Bates PE, Cuvo T, Miner CA, Korabek CA (2001) Simulated and community-based instruction involving persons with mild and moderate mental retardation. Res Dev Disabil 22: 95-115.

18. Baylis P, Snowling MJ (2012) Evaluation of a phonological reading programme for children with Down syndrome. Child Lang Teach Ther 28: 39-56.

19. Begeer S, Gevers C, Clifford P, Verhoeve M, Kat K, et al. (2011) Theory of mind training in children with autism: A randomized controlled trial. J Autism Dev Disord 41: 997-1006.

20. Ben Itzchak E, Zachor DA (2011) Who benefits from early intervention in autism spectrum disorders? Res Autism Spectr Disord 5: 345-350.

21. Ben-Itzchak E, Zachor DA (2007) The effects of intellectual functioning and autism severity on outcome of early behavioral intervention for children with autism. Res Dev Disabil 28: 287-303.

22. Bendixen RM, Elder JH, Donaldson S, Kairalla JA, Valcante G, et al. (2011) Effects of a father-based in-home intervention on perceived stress and family dynamics in parents of children with autism. Am J Occup Ther 65: 679-687.

23. Bilgin S, Gozum S (2009) Reducing burnout in mothers with an intellectually disabled child: an education programme. J Adv Nurs. pp. 2552-2561.

24. Bird EK, Gaskell A, Babineau MD, Macdonald S (2000) Novel word acquisition in children with Down syndrome: does modality make a difference? J Commun Disord 33: 241-265.

25. Brandao Coutinho MT (2004) Supporting the family and parental training. Analise Psicologica 22: 55-64.

26. Bruce M, Collins S, Langdon P, Powlitch S, Reynolds S (2010) Does training improve understanding of core concepts in cognitive behaviour therapy by people with intellectual disabilities? A randomized experiment. Brit J Clin Psychol: 1-13.

27. Carr D, Felce J (2007) Brief report: Increase in production of spoken words in some children with autism after PECS teaching to Phase III. J Autism Dev Disord 37: 780-787.

28. Carter AS, Messinger DS, Stone WL, Celimli S, Nahmias AS, et al. (2011) A randomized controlled trial of Hanen's 'More Than Words' in toddlers with early autism symptoms. J Child Psychol Psychiatry 52: 741-752.

29. Chadwick O, Momcilocic N, Rossiter R, Stumbles E, Taylor E (2001) A randomized trial of brief individual versus group parent training for behaviour problems in children with severe learning disabilities. Behav Cogn Psychother 29: 151-167.

30. Chung KKH, Tam YH (2005) Effects of cognitive-based instruction on mathematical problem solving by learners with mild intellectual disabilities. J Intellect Dev Disabil 30: 207-216.

31. Ciechomski LD, Jackson KL, Tonge B, King NJ, Heyne DA (2001) Intellectual disability and anxiety in children: A group-based parent skills-training intervention. Behav Change 18: 204-212.

32. Conner NW, Fraser MW (2011) Preschool Social–Emotional Skills Training: A Controlled Pilot Test of the Making Choices and Strong Families Programs. Res Soc Work Pract 21: 699-711.

33. Conners F, Rosenquist C, Arnett L, Moore M, Hume L (2008) Improving memory span in children with Down syndrome. J Intellect Disabil Res 52: 244-255.

34. Coughlin M, Sharry J, Fitzpatrick C, Guerin S, Drumm M (2009) A controlled clinical evaluation of the parents plus children's programme: a video-based programme for parents of children aged 6 to 11 with behavioural and developmental problems. Clin Child Psychol Psychiatry 14: 541-558.

35. Cowen PS, Reed DA (2002) Effects of respite care for children with developmental disabilities: evaluation of an intervention for at risk families. Public Health Nurs 19: 272-283.

36. Crites SA, Dunn C (2004) Teaching Social Problem Solving to Individuals with Mental Retardation. Educ Train Dev Disabil 39: 301-309.

37. de la Iglesia J, Buceta M, Campos A (2005) Prose learning in children and adults with Down syndrome: The use of visual and mental image strategies to improve recall. J Intellect Dev Disabil 30: 199-206.

38. Demers J, French DC, Moore D (2000) The preliminary evaluation of a program to help educators address the substance use/prevention needs of special students. J Alcohol Drug Educ 46: 14-26.

39. Dessemontet R, Bless G, Morin D (2012) Effects of inclusion on the academic achievement and adaptive behaviour of children with intellectual disabilities. J Intellect Disabil Res 56: 579-587.

40. Drew A, Baird G, Baron-Cohen A, Cox A, Slonims V, et al. (2002) A pilot randomised control trial of a parent training intervention for pre-school children with autism. Eur Child Adolesc Psychiatry 11: 266-272.

41. Drummond J, Fleming D, McDonald L, Kysela GM (2005) Randomized Controlled Trial of a Family Problem-Solving Intervention. Clin Nurs Res 14: 57-80.

42. Drysdale J, Casey J, Porter-Armstrong A (2008) Effectiveness of training on the community skills of children with intellectual disabilities. Scand J Occup Ther 15: 247-255.

43. Eikeseth S, Hayward D, Gale C, Gitlesen J-P, Eldevik S (2009) Intensity of supervision and outcome for preschool aged children receiving early and intensive behavioral interventions: A preliminary study. Res Autism Spectr Disord 3: 67-73.

44. Eldevik S, Eikeseth S, Jahr E, Smith T (2006) Effects of Low-Intensity Behavioral Treatment for Children with Autism and Mental Retardation. J Autism Dev Disord 36: 211-224.

45. Eldevik S, Hastings RP, Jahr E, Hughes J (2012) Outcomes of behavioral intervention for children with autism in mainstream pre-school settings. J Autism Dev Disord 42: 210-220.

46. Elosua MR, Garcia-Madruga JA, Gutierrez F, Luque JL, Garate M (2002) Effects of an intervention in active strategies for text comprehension and recall. Span J Psychol 5: 90-101.

47. Erguner-Tekinalp B, Akkok F (2004) The effects of a coping skills training program on the coping skills, hopelessness, and stress levels of mothers of children with autism. Int J Adv Couns 26: 257-269.

48. Fabio RA, Giannatiempo S, Oliva P, Murdaca AM (2011) The increase of attention in Rett Syndrome: A pre-test/post-test research design. J Dev Phys Disabil 23: 99-111.

49. Farber MLZ, Maharaj R (2005) Empowering high-risk families of children with disabilities. Res Soc Work Pract 15: 501-515.

50. Fava L, Strauss K, Valeri G, D'Elia L, Arima S, et al. (2011) The effectiveness of a cross-setting complementary staff- and parent-mediated early intensive behavioral intervention for young children with ASD. Res Autism Spectr Disord 5: 1479-1492.

51. Feldman MA, Werner SE (2002) Collateral effects of behavioral parent training on families of children with developmental disabilities and behavior disorders. Behav Interv 17:. 75-83.

52. Fennick E, Royle J (2003) Community inclusion for children and youth with developmental disabilities. Focus Autism Other Dev Disabl 18: 20-27.

53. Fernell E, Hedvall A, Westerlund J, Carlsson LH, Eriksson M, et al. (2011) Early intervention in 208 Swedish preschoolers with autism spectrum disorder. A prospective naturalistic study. Res Dev Disabil 32: 2092-2101.

54. Fey ME, Warren SF, Brady N, Finestack LH, Bredin-Oja SL, et al. (2006) Early effects of responsivity education/prelinguistic milieu teaching for children with developmental delays and their parents. J Speech Lang Hear Res 49: 526-547.

55. Flanagan HE, Perry A, Freeman NL (2012) Effectiveness of large-scale community-based intensive Behavioral Intervention: A waitlist comparison study exploring outcomes and predictors. Res Autism Spectr Disord 6: 673-682.

56. Flores MM, Nelson C, Hinton V, Franklin TM, Strozier SD, et al. (2013) Teaching reading comprehension and language skills to students with autism spectrum disorders and developmental disabilities using direct instruction. Educ Train Autism Dev Disabil 48: 41-48.

57. Ganesh Kumar S, Shekhar A, Unnikrishnan B, Kotian MS (2011) Effect of psychosocial intervention on quality of life and disability grading of mentally disabled adolescents. Curr Pediatr Res 15: 127-131.

58. Gaunt L, Moni KB, Jobling A (2012) Developing numeracy in young adults with Down syndrome: A preliminary investigation of specific teaching strategies. J Dev Disabil 18: 10-25.

59. Giarelli E, Souders M, Pinto-Martin J, Bloch J, Levy SE (2005) Intervention pilot for parents of children with autistic spectrum disorder. Pediatr Nurs 31: 389-399.

60. Girolametto L, Weitzman E, Clements-Baartman J (1998) Vocabulary intervention for children with Down syndrome: parent training using focused stimulation. Infant-Toddler Interv 8: 109-125.

61. Godfrey J, Pring T, Gascoigne M (2005) Developing children's conversational skills in mainstream schools: an evaluation of group therapy. Child Lang Teach Ther 21: 251-261.

62. Golnik A, Scal P, Wey A, Gaillard P (2012) Autism-specific primary care medical home intervention. J Autism Dev Disord 42: 1087-1093.

63. Goods KS, Ishijima E, Chang Y-C, Kasari C (2013) Preschool based JASPER intervention in minimally verbal children with autism: Pilot RCT. J Autism Dev Disord 43: 1050-1056.

64. Gordon K, Pasco G, McElduff F, Wade A, Howlin P, et al. (2011) A communication-based intervention for nonverbal children with autism: What changes? Who benefits? J Consult Clin Psychol 79: 447-457.

65. Gore N, Umizawa H (2011) Challenging behavior training for teaching staff and family carers of children with intellectual disabilities: A preliminary evaluation. J Policy Prac in Intellect Disabil 8: 266-275.

66. Granat T, Nordgren I, Rein G, Sonnander K (2012) Group intervention for siblings of children with disabilities: A pilot study in a clinical setting. Disabil Rehabil 34: 69-75.

67. Green J, Charman T, Mc Conachie H, Aldred C, Slonims V, et al. (2011) Parent-mediated communication-focused treatment for preschool children with autism (MRC PACT); a randomised controlled trial. Eur Psychiatry 26: 1897.

68. Green J, Charman T, McConachie H, Aldred C, Slonims V, et al. (2010) Parent-mediated communication-focused treatment in children with autism (PACT): A radomised controlled trial. Lancet 375: 2152-2160.

69. Gulsrud AC, Kasari C, Freeman S, Paparella T (2007) Children with autism's response to novel stimuli while participating in interventions targeting joint attention or symbolic play skills. Autism 11: 535-546.

70. Guralnick MJ, Connor RT, Neville B, Hammond MA (2006) Promoting the Peer-Related Social Development of Young Children With Mild Developmental Delays: Effectiveness of a Comprehensive Intervention. Am J Ment Retard 111: 336-356.

71. Hagiliassis N, Gulbenkoglu H, Di Marco M, Young S, Hudson A (2005) The Anger Management Project: a group intervention for anger in people with physical and multiple disabilities. J Intellect Dev Disabil 30: 86-96.

72. Hagner D, Kurtz A, Cloutier H, Arakelian C, Brucker DL, et al. (2012) Outcomes of a family-centered transition process for students with autism spectrum disorders. Focus Autism Other Dev Disabil 27: 42-50.

73. Holtz CA, Carrasco JM, Mattek RJ, Fox RA (2009) Behavior problems in toddlers with and without developmental delays: comparison of treatment outcomes. Child Fam Behav Ther 31: 292-311.

74. Hong C, Lee I (2012) Effects of neurofeedback training on attention in children with intellectual disability. J Neurother 16: 110-122.

75. Hopkins IM, Gower MW, Perez TA, Smith DS, Amthor FR, et al. (2011) Avatar assistant: Improving social skills in students with an ASD through a computer-based intervention. J Autism Dev Disord 41: 1543-1555.

76. Howlin P, Gordon R, Pasco G, Wade A, Charman T (2007) The effectiveness of Picture Exchange Communication System (PECS) training for teachers of children with autism: A pragmatic, group randomised controlled trial. J Child Psychol Psychiatry 48: 473-481.

77. Hu J, Lin JD, Yen CF, Loh CH, Hsu SW, et al. (2010) Effectiveness of a stress-relief initiative for primary caregivers of adolescents with intellectual disability. J Intellect Dev Disabil 35: 29-35.

78. Hudson A, Reece J, Cameron C, Matthews J (2009) Effects of child characteristics on the outcomes of a parent support program. J Intellect Dev Disabil 34: 123-132.

79. Hudson AM, Matthews JM, Gavidia-Payne ST, Cameron CA, Mildon RL, et al. (2003) Evaluation of an intervention system for parents of children with intellectual disability and challenging behaviour. J Intellect Disabil Res 47(pt 4/5): 238-249.

80. Ingersoll B (2010) Brief report: Pilot randomized controlled trial of reciprocal imitation training for teaching elicited and spontaneous imitation to children with autism. J Autism Dev Disord 40: 1154-1160.

81. Ingersoll B (2012) Effect of a focused imitation intervention on social functioning in children with autism. J Autism Dev Disord 42: 1768-1773.

82. Itzchak EB, Zachor DA (2009) Change in autism classification with early intervention: Predictors and outcomes. Res Autism Spectr Disord 3: 967-976.

83. Jarusiewicz B (2002) Efficacy of Neurofeedback for Children in the Autistic Spectrum: A Pilot Study. J Neurother 6: 39-49.

84. Jones EA, Carr EG, Feeley KM (2006) Multiple Effects of Joint Attention Intervention for Children With Autism. Behav Modif 30: 782-834.

85. Josman N, Ben-Chaim HM, Friedrich S, Weiss PL (2008) Effectiveness of virtual reality for teaching street-crossing skills to children and adolescents with autism. Int J Disabil Hum Dev 7: 49-56.

86. Kaiser AP, Roberts MY (2013) Parent-implemented enhanced milieu teaching with preschool children who have intellectual disabilities. J Speech Lang Hear Res 56: 295-309.

87. Kalyva E, Avramidis E (2005) Improving Communication Between Children with Autism and Their Peers Through the 'Circle of Friends': A Small-scale Intervention Study. J Appl Res Intellect Disabil 18: 253-261.

88. Kamps DM, Dugan EP, Leonard BR, Daoust PM (1994) Enhanced small group instruction using choral responding and student interaction for children with autism and developmental disabilities. Am J Ment Retard 99: 60-73.

89. Kamps DM, Leonard BR, Vernon S, Dugan EP, Delquadri JC, et al. (1992) Teaching social skills to students with autism to increase peer interactions in an integrated first-grade classroom. J Appl Behav Anal 25: 281-288.

90. Kasari C, Freeman S, Paparella T (2006) Joint attention and symbolic play in young chidlren with autism: A randomized controlled intervention study. J Child Psychol Psychiatry 47: 611-620.

91. Kasari C, Gulsrud AC, Wong C, Kwon S, Locke J (2010) Randomized controlled caregiver mediated joint engagement intervention for toddlers with autism. J Autism Dev Disord 40: 1045-1056.

92. Kasari C, Rotheram-Fuller E, Locke J, Gulsrud A (2012) Making the connection: Randomized controlled trial of social skills at school for children with autism spectrum disorders. J Child Psychol Psychiatry 53: 431-439.

93. Keen D, Couzens D, Muspratt S, Rodger S (2010) The effects of a parent-focused intervention for children with a recent diagnosis of autism spectrum disorder on parenting stress and competence. Res Autism Spectr Disord 4: 229-241.

94. Keen D, Rodger S, Doussin K, Braithwaite M (2007) A pilot study of the effects of a social-pragmatic intervention on the communication and symbolic play of children with autism. Autism 11: 63-71.

95. Kemp C (2003) Investigating the Transition of Young Children With Intellectual Disabilities to Mainstream Classes: An Australian Perspective. Int J Disabil Dev Educ 50: 403-433.

96. Kim JM, Mahoney G (2005) The effects of relationship focused intervention on Korean parents and their young children with disabilities. Res Dev Disabil 26: 117-130.

97. Kirkham MA (1993) Two-year follow-up of skills training with mothers of children with disabilities. Am J Ment Retard 97: 509-520.

98. Kleefman M, Jansen DEMC, Reijneveld SA (2011) The effectiveness of Stepping Stones Triple P: the design of a randomised controlled trial on a parenting programme regarding children with mild intellectual disability and psychosocial problems versus care as usual. BMC Public Health 11: 676.

99. Kleve L, Crimlisk S, Shoebridge P, Greenwood R, Baker B, et al. (2011) Is the Incredible Years programme effective for children with neuro-developmental disorders and for families with Social Services involvement in the "real world" of community CAMHS? Clini Child Psychol Psychiatry 16: 253-264.

100. Klintwall L, Gillberg C, Bolte S, Fernell E (2012) The efficacy of intensive behavioral intervention for children with autism: A matter of allegiance? J Autism Dev Disord 42: 139-140.

101. Kobak KA, Stone WL, Wallace E, Warren Z, Swanson A, et al. (2011) A Web-based tutorial for parents of young children with autism: Results from a pilot study. Telemed E Health 17: 804-808.

102. Konstantareas M, Rios A, Ramnarace C (2010) Intensive Behavioural Intervention (IBI) training: Cooperation and its relationship to language and social competence in children with Autism Spectrum Disorder (ASD). J Dev Disabil 16: 67-68.

103. Kroeger K, Schultz JR, Newsom C (2007) A comparison of two group-delivered social skills programs for young children with autism. J Autism Dev Disord 37: 808-817.

104. Kroesbergen EH, Van Luit JE (2005) Constructivist mathematics education for students with mild mental retardation. Eur J Spec Needs Educ 20: 107-116.

105. Kurani D, Nerurkar A, Miranda L, Jawadwala F, Prabhulkar D (2009) Impact of parents' involvement and engagement in a learning readiness programme for children with severe and profound intellectual disability and complex needs in India. J Intellect Disabil 13: 269-289.

106. Landa RJ, Holman KC, O'Neill AH, Stuart EA (2011) Intervention targeting development of socially synchronous engagement in toddlers with autism spectrum disorder: A randomized controlled trial. J Child Psychol Psychiatry 52: 13-21.

107. Lawton K, Kasari C (2012) Brief report: Longitudinal improvements in the quality of joint attention in preschool children with autism. J Autism Dev Disord 42: 307-312.

108. Lecas J-F, Mazaud A-M, Reibel E, Rey A (2011) Using visual strategies to support verbal comprehension in an adolescent with Down syndrome. Child Lang Teach Ther 27: 84-96.

109. Lemons CJ, Fuchs D (2010) Modeling response to reading intervention in children with Down syndrome: An examination of predictors of differential growth. Read Res Q 45: 134-168.

110. Leung C, Fan A, Sanders MR (2013) The effectiveness of a Group Triple P with Chinese parents who have a child with developmental disabilities: A randomized controlled trial. Res Dev Disabil 34: 976-984.

111. Lobato DJ, Kao BT (2005) Brief report: Family-based group intervention for young siblings of children with chronic illness and developmental disability. J Pediatr Psychol 30: 678-682.

112. Loumidis KS, Hill A (1997) Training social problem-solving skill to reduce maladaptive behaviours in intellectual disability groups: the influence of individual difference factors. J Appl Res Intellect Disabil 10: 217-237.

113. Lucas P, Liabo K, Roberts H (2002) Do behavioural treatments for sleep disorders in children with Down's syndrome work? Arch Dis Child 87: 413-414.

114. Lundberg I, Reichenberg M (2013) Developing reading comprehension among students with mild intellectual disabilities: An intervention study. Scan J Educ Res 57: 89-100.

115. Magiati I, Charman T, Howlin P (2007) A two-year prospective follow-up study of community-based early intensive behavioural intervention and specialist nursery provision for children with autism spectrum disorders. J Child Psychol Psychiatry 48: 803-812.

116. Magiati I, Moss J, Charman T, Howlin P (2011) Patterns of change in children with autism spectrum disorders who received community based comprehensive interventions in their pre-school years: A seven year follow-up study. Res Autism Spectr Disord 5: 1016-1027.

117. May FS, McLean LA, Anderson A, Hudson A, Cameron C, et al. (2013) Father participation with mothers in the Signposts program: an initial investigation. J Intellect Dev Disabil 38: 39-47.

118. McConkey R, Truesdale-Kennedy M, Crawford H, McGreevy E, Reavey M, et al. (2010) Preschoolers with autism spectrum disorders: Evaluating the impact of a home-based intervention to promote their communication. Early Child Dev Care 180: 299-315.

119. McIntyre LL (2008) Parent training for young children with developmental disabilities: Randomized controlled trial. Am J Ment Retard 113: 356-368.

120. Montgomery P, Stores G, Wiggs L (2004) The relative efficacy of two brief treatments for sleep problems in young learning disabled (mentally retarded) children: a randomised controlled trial. Arch Dis Child 89: 125-130.

121. Motsch H-J, Ulrich T (2012) Effects of the strategy therapy ‘lexicon pirate’ on lexical deficits in preschool age: A randomized controlled trial. Child Lang Teach Ther 28: 159-175.

122. Mullins LL, Aniol K, Boyd ML, Page MC, Chaney JM (2002) The influence of respite care on psychological distress in parents of children with developmental disabilities: a longitudinal study. Child Serv Soc Pol Res Prac 5: 123-138.

123. Narramore N (2008) Meeting the emotional needs of parents who have a child with complex needs. J Child Young People Nurse 2: 103-107.

124. Nixon CD, Singer GH (1993) Group cognitive-behavioral treatment for excessive parental self-blame and guilt. Am J Ment Retard 97: 665-672.

125. Noone SJ, Hastings RP (2009) Building psychological resilience in support staff caring for people with intellectual disabilities: pilot evaluation of an acceptance-based intervention. J Intellect Dis 13: 43-53.

126. O'Connor C, Stagnitti K (2011) Play, behaviour, language and social skills: The comparison of a play and a non-play intervention within a specialist school setting. Res Dev Disabil 32: 1205-1211.

127. Okuno H, Nagai T, Sakai S, Mohri I, Yamamoto T, et al. (2011) Effectiveness of modified parent training for mothers of children with Pervasive Developmental Disorder on parental confidence and children's behavior. Brain Dev 33: 152-160.

128. Olivar-Parra J-S, De-La-Iglesia-Gutierrez M, Forns M (2011) Training referential communicative skills to individuals with autism spectrum disorder: a pilot study. Psychol Rep 109: 921-939.

129. Oliver PC, Piachaud J, Tyrer P, Regan A, Dack M, et al. (2005) Randomized controlled trial of assertive community treatment in intellectual disability: the TACTILD study. J Intellect Disabil Res 49: 507-515.

130. Oosterling I, Visser J, Swinkels S, Rommelse N, Donders R, et al. (2010) Randomized controlled trial of the focus parent training for toddlers with autism: 1-year outcome. J Autism Dev Disord 40: 1447-1458.

131. Ozonoff S, Miller J (1995) Teaching theory of mind: A new approach to social skills training for individuals with autism. J Autism Dev Disord 25: 415-433.

132. Pajareya K, Nopmaneejumruslers K (2011) A pilot randomized controlled trial of DIR/FloortimeTM parent training intervention for pre-school children with autistic spectrum disorders. Autism 15: 563-577.

133. Palmer SB, Wehmeyer ML, Gipson K, Agran M (2004) Promoting Access to the General Curriculum by Teaching Self-Determination Skills. Except Child 70: 427-439.

134. Panerai S, Ferrante L, Zingale M (2002) Benefits of the Treatment and Education of Austistic and Comunication Handicapped Children (TEACCH) programme as compared with a non-specific approach. J Intellect Disabil Res 46: 318-327.

135. Pelchat D, Bisson J, Ricard N, Perreault M, Bouchard JM (1999) Longitudinal effects of an early family intervention programme on the adaptation of parents of children with a disability. Int J Nurs Stud 36: 465-477.

136. Pelchat D, Ricard N, Lefebvre H (2001) [Parents' adaptation to a delayed developmental child. Effects of a family early intervention program]. Infirm Que 9: 14-24.

137. Perez LM, Garcia EG (2002) Programme for the improvement of metamemory in people with medium and mild mental retardation. Psychol in Spain 6: 96-101.

138. Perry A, Condillac RA (2010) The TRE-ADD preschool parent training program: Program evaluation of an innovative service delivery model. J Dev Disabil 16: 8-16.

139. Perry A, Cummings A, Geier JD, Freeman NL, Hughes S, et al. (2008) Effectiveness of Intensive Behavioral Intervention in a large, community-based program. Res Autism Spectr Disord 2: 621-642.

140. Perry A, Cummings A, Geier JD, Freeman NL, Hughes S, et al. (2011) Predictors of outcome for children receiving intensive behavioral intervention in a large, community-based program. Res Autism Spectr Disord 5: 592-603.

141. Piazza CC, Fisher WW, Sherer M (1997) Treatment of multiple sleep problems in children with developmental disabilities: faded bedtime with response cost versus bedtime scheduling. Dev Med Child Neurol 39: 414-418.

142. Pillay M, Alderson-Day B, Wright B, Williams C, Urwin B (2011) Autism Spectrum Conditions-Enhancing Nurture and Development (ASCEND): An evaluation of intervention support groups for parents. Clin Child Psychol Psychiatry 16: 5-20.

143. Pineda J, Brang D, Hecht E, Edwards L, Carey S, et al. (2008) Positive behavioral and electrophysiological changes following neurofeedback training in children with autism. Res Autism Spectr Disord 2: 557-581.

144. Puttahraksa P, Tilokskulchai F, Sitthimongkol Y, Prasopkittikul T, Liknapichitkul D (2006) Empowerment program on promoting perceived self-efficacy in caregivers of autistic children. Thai J Nurse Res 10: 180-190.

145. Quinn M, Carr A, Carroll, O'Sullivan D (2007) Parents Plus program 1: evaluation of its effectiveness for pre-school children with developmental disabilities and behavioural problems. J Appl Res Intellect Disabil. pp. 345-359.

146. Quirmbach LM, Lincoln AJ, Feinberg-Gizzo MJ, Ingersoll BR, Andrews SM (2009) Social stories: mechanisms of effectiveness in increasing game play skills in children diagnosed with autism spectrum disorder using a pretest posttest repeated measures randomized control group design. J Autism Dev Disord 39: 299-321.

147. Raghavan R, Newell R, Waseem F, Small N (2009) A randomized controlled trial of a specialist liaison worker model for young people with intellectual disabilities with challenging behaviour and mental health needs. J Appl Res Intellect Disabil 22: 256-263.

148. Reed P, Osborne LA, Corness M (2010) Effectiveness of special nursery provision for children with autism spectrum disorders. Autism 14: 67-82.

149. Reed P, Osborne LA, Makrygianni M, Waddington E, Etherington A, et al. (2013) Evaluation of the Barnet Early Autism Model (BEAM) teaching intervention programme in a 'real world' setting. Res Autism Spect Disord 7: 631-638.

150. Reid MJ, Webster-Stratton C, Hammond M (2007) Enhancing a classroom social competence and problem-solving curriculum by offering parent training to families of moderate- to high-risk elementary school children. J Clin Child Psychol 36: 605-620.

151. Rickards A, Walstab J, Wright-Rossi R, Simpson J, Reddihough D (2009) One-year follow-up of the outcome of a randomized controlled trial of a home-based intervention programme for children with autism and developmental delay and their families. Child Health Care Dev 35: 593-602.

152. Rickards AL, Walstab JE, Wright-Rossi RA, Simpson J, Reddihough DS (2007) A randomized, controlled trial of a home-based intervention program for children with autism and developmental delay. J Dev Behav Pediatr 28: 308-316.

153. Rocha ML, Schreibman L, Stahmer AC (2007) Effectiveness of training parents to teach joint attention in children with autism. J of Early Interv 29: 154-172.

154. Roeyers H (1996) The influence of nonhandicapped peers on the social interactions of children with a pervasive development disorder. J Autism Dev Disord 26: 303-320.

155. Rogers SJ, Estes A, Lord C, Vismara L, Winter J, et al. (2012) Effects of a brief Early Start Denver model (ESDM)-based parent intervention on toddlers at risk for autism spectrum disorders: a randomized controlled trial. J Am Acad Child Adolesc Psychiatry 51: 1052-1065.

156. Russell PSS, John JK, Lakshmanan J, Russell S, Lakshmidevi KM (2004) Family intervention and acquisition of adaptive behaviour among intellectually disabled children. J Intellect Disabil 8: 383-395.

157. Ryan C, Charragain CN (2010) Teaching emotion recognition skills to children with autism. J Autism Dev Disord 40: 1505-1511.

158. Sallows GO, Graupner TD (2005) Intensive behavioral treatment for children with autism: Four-year outcome and predictors. Am J Ment Retard 110: 417-438.

159. Salt J, Shemilt J, Sellars V, Boyd S, Coulson T, et al. (2002) The Scottish Centre for Autism preschool treatment programme. Autism 6: 33-46.

160. Samadi SA, McConkey R, Kelly G (2013) Enhancing parental well-being and coping through a family-centred short course for Iranian parents of children with an autism spectrum disorder. Autism 17: 27-43.

161. Sanz MT, Menendez J (2010) Parents' training: Effects of the self-help skills programme with down's syndrome babies. Early Child Dev Care 180: 735-742.

162. Saravanan C, Rangaswamy K (2012) Effectiveness of counselling on the attitudes of mothers towards their children with intellectual disability. Asia Pac J Counsel Psychother 3: 82-94.

163. Schertz HH, Odom SL, Baggett KM, Sideris JH (2013) Effects of Joint Attention Mediated Learning for toddlers with autism spectrum disorders: An initial randomized controlled study. Early Child Res Q 28: 249-258.

164. Schultz Cynthia L, Schultz Noel C, Bruce Elizabeth J, Smyrnios Kosmas X, et al. (1993) Psychoeducational support for parents of children with intellectual disability: An outcome study. Int J Disabil Dev Educ 40: 205-216.

165. Schwartz IS, Sandall SR, McBride BJ, Boulware G-L (2004) Project DATA (Developmentally Appropriate Treatment for Autism): An Inclusive School-Based Approach to Educating Young Children with Autism. Topics Early Child Spec Educ 24: 156-168.

166. Self T, Scudder RR, Weheba G, Crumrine D (2007) A virtual approach to teaching safety skills to children with autism spectrum disorder. Topics Lang Disord 27: 242.

167. Sheinkopf SJ, Siegel B (1998) Home-based behavioral treatment of young children with autism. J Autism Dev Disord 28: 15-23.

168. Shu BC, Lung FW, Huang C (2002) Mental health of primary family caregivers with children with intellectual disability who receive a home care programme. J Intellect Disabil Res 46: 257-263.

169. Siller M, Hutman T, Sigman M (2013) A parent-mediated intervention to increase responsive parental behaviors and child communication in children with ASD: A randomized clinical trial. J Autism Dev Disord 43: 540-555.

170. Sofronoff K, Farbotko M (2002) The effectiveness of parent management training to increase self-efficacy in parents of children with Asperger syndrome. Autism 6: 271-286.

171. Sofronoff K, Jahnel D, Sanders M (2011) Stepping Stones Triple P seminars for parents of a child with a disability: a randomized controlled trial. Res Dev Disabil 32: 2253-2262.

172. Sofronoff K, Leslie A, Brown W (2004) Parent management training and Asperger syndrome: a randomized controlled trial to evaluate a parent based intervention. Autism 8: 301-317.

173. Solomon R, Necheles J, Ferch C, Bruckman D (2007) Pilot study of a parent training program for young children with autism: The PLAY Project Home Consultation program. Autism 11: 205-224.

174. Stichter JP, O'Connor KV, Herzog MJ, Lierheimer K, McGhee SD (2012) Social competence intervention for elementary students with Aspergers syndrome and high functioning autism. J Autism Dev Disord 42: 354-366.

175. Stores R, Stores G (2004) Evaluation of Brief Group-Administered Instruction for Parents to Prevent or Minimize Sleep Problems in Young Children with Down Syndrome. J Appl Res Intellect Disabil 17: 61-70.

176. Strain PS, Bovey EH, II (2011) Randomized, controlled trial of the LEAP model of early intervention for young children with autism spectrum disorders. Topics Early Child Spec Educ 31: 133-154.

177. Strauss K, Vicari S, Valeri G, D'Elia L, Arima S, et al. (2012) Parent inclusion in early intensive behavioral intervention: The influence of parental stress, parent treatment fidelity and parent-mediated generalization of behavior targets on child outcomes. Res Dev Disabil 33: 688-703.

178. Tang M-H, Lin C-K, Lin W-H, Chen C-H, Tsai S-W, et al. (2011) The effect of adding a home program to weekly institutional-based therapy for children with undefined developmental delay: a pilot randomized clinical trial. J Chin Med Assoc 74: 259-266.

179. Tannock R, Girolametto L, Siegel LS (1992) Language intervention with children who have developmental delays: effects of an interactive approach. Am J Ment Retard 97: 145-160.

180. Taylor JL, Novaco RW, Gillmer BT, Robertson A, Thorne I (2005) Individual cognitive-behavioural anger treatment for people with mild-borderline intellectual disabilities and histories of aggression: a controlled trial. Brit J Clin Psychol 44: 367-382.

181. Temple K (2007) A randomized comparison of the effect of two prelinguistic communication interventions on the acquisition of spoken communication in preschoolers with ASD. Child Care Health Dev 33: 348-349.

182. Todd S, Bromley J, Ioannou K, Harrison J, Mellor C, et al. (2010) Using Group-Based Parent Training Interventions with Parents of Children with Disabilities: A Description of Process, Content and Outcomes in Clinical Practice. Child Adol Ment Health 15: 171-175.

183. Tonge B, Brereton A, Kiomall M, Mackinnon A, King N, et al. (2006) Effects on parental mental health of an education and skills training program for parents of young children with autism: a randomized controlled trial. J Am Acad Child Adolesc Psychiatry 45: 561-569.

184. van Bysterveldt AK, Gillon GT, Moran C (2006) Enhancing Phonological Awareness and Letter Knowledge in Preschool Children with Down Syndrome. Int J Disabil Dev Educ 53: 301-329.

185. Van Der Bijl C, Alant E, Lloyd L (2006) A comparison of two strategies of sight word instruction in children with mental disability. Res Dev Disabil 27: 43-55.

186. Venker CE, McDuffie A, Weismer SE, Abbeduto L (2012) Increasing verbal responsiveness in parents of children with autism:A pilot study. Autism 16: 568-585.

187. Vismara LA, Colombi C, Rogers SJ (2009) Can one hour per week of therapy lead to lasting changes in young children with autism? Autism 13: 93-115.

188. Vismara LA, Rogers SJ (2008) The Early Start Denver Model: a case study of an innovative practice. J Early Interv 31: 91-108.

189. Wang P (2008) Effects of a parent training program on the interactive skills of parents of children with autism in China. J Pol Prac Intellect Disabil 5: 96-104.

190. Warren SF, Fey ME, Finestack LH, Brady NC, Bredin-Oja SL, et al. (2008) A randomized trial of longitudinal effects of low-intensity responsivity education/prelinguistic milieu teaching. J Speech Lang Hear Res 51: 451-470.

191. Wetherby AM, Woods JJ (2006) Early Social Interaction Project for Children With Autism Spectrum Disorders Beginning in the Second Year of Life: A Preliminary Study. Topics Early Child Spec Educ 26: 67-82.

192. Whittingham K, Sofronoff K, Sheffield J, Sanders MR (2009) Stepping Stones Triple P: An RCT of a parenting program with parents of a child diagnosed with an autism spectrum disorder. J Abnorm Child Psychol 37: 469-480.

193. Wiggs L, Stores G (1999) Behavioural treatment for sleep problems in children with severe learning disabilities and challenging daytime behaviour: effect on daytime behaviour. J Child Psychol Psychiatry 40: 627-635.

194. Wiggs L, Stores G (2001) Behavioural treatment for sleep problems in children with severe intellectual disabilities and daytime challenging behaviour: effect on mothers and fathers. Brit J Health Psychol 6: 257-269.

195. Williams BT, Gray KM, Tonge BJ (2012) Teaching emotion recognition skills to young children with autism: a randomised controlled trial of an emotion training programme. J Child Psychol Psychiatry 53: 1268-1276.

196. Wong FKD, Poon A (2010) Cognitive behavioural group treatment for Chinese parents with children with developmental disabilities in Melbourne, Australia: an efficacy study. Aust N Z J Psychiatry 44: 742-749.

197. Wong SY, Lai AC, Martinson I, Wong TKS (2006) Effects of an education programme on family participation in the rehabilitation of children with developmental disability. J Intellect Disabil 10: 165-189.

198. Yildirim A, Asilar RH, Karakurt P (2013) Effects of a nursing intervention program on the depression and perception of family functioning of mothers with intellectually disabled children. J Clin Nurse 22: 251-261.

199. Zachor DA, Ben-Itzchak E, Rabinovich A-L, Lahat E (2007) Change in autism core symptoms with intervention. Res Autism Spectr Disord 1: 304-317.

200. Zachor DA, Itzchak EB (2010) Treatment approach, autism severity and intervention outcomes in young children. Res Autism Spectr Disord 4: 425-432.
